# Silk Fibroin Counteracts Fibroblast Senescence to Restore ECM Homeostasis in Aged Skin

**DOI:** 10.1016/j.bioactmat.2025.12.006

**Published:** 2026-01-06

**Authors:** Jialing Cheng, Guo Bao, Demin Lin, Hongliang Wang, Yanfang Yang, Youbai Chen, Meiying Ning, Jun Ye, Yuling Liu

**Affiliations:** a State Key Laboratory of Bioactive Substance and Function of Natural Medicines, Institute of Materia Medica, Chinese Academy of Medical Sciences & Peking Union Medical College, Beijing, P.R. China; b Beijing Key Laboratory of Drug Delivery Technology and Novel Formulation, Institute of Materia Medica, Chinese Academy of Medical Sciences & Peking Union Medical College, Beijing, P.R. China; c National Research Institute for Family Planning, Beijing, P.R. China; d Department of Plastic and Reconstructive Surgery, Chinese PLA General Hospital, Beijing, P.R. China

**Keywords:** Silk fibroin, Skin aging, Extracellular matrix homeostasis, Senescence-associated secretory phenotype, MAPK/AP-1/MMP pathway, Fibroblast rejuvenation

## Abstract

Skin aging is characterized by a progressive decline in regenerative capacity, primarily driven by fibroblast senescence, oxidative stress, chronic inflammation, and the degradation of type I/III collagen, culminating in an extracellular matrix (ECM) imbalance. Current injectable fillers—such as hyaluronic acid, collagen, and PLLA—provide temporary structural support but fail to address the underlying cellular senescence or restore ECM homeostasis, highlighting the need for regenerative biomaterials. Silk fibroin (SF), a natural protein, self-assembles into a β-sheet-rich scaffold that structurally supports fibroblasts in depositing collagen and elastin, thereby improving the skin's ECM, accelerating wound healing, and promoting tissue regeneration. However, its role in modulating fibroblast senescence and ECM remodeling remains unclear. This study demonstrates that SF provides a suitable microenvironment for the adhesion and proliferation of fibroblasts, reducing the accumulation of SASP factors and facilitating the transition of fibroblasts from a senescent to a functional state. Furthermore, SF improves the skin microenvironment by reducing reactive oxygen species (ROS) and matrix metalloproteinase (MMP) expression through modulation of the ROS–MAPK–AP-1–MMP signal pathway, thereby delaying collagen degradation in aged skin. These findings reveal that SF uniquely rejuvenates fibroblasts and restores ECM homeostasis through a non-inflammatory mechanism, distinguishing it from conventional fillers that rely on inflammatory pathways for collagen induction. This work establishes SF as a next-generation injectable biomaterial with dual targeting of cellular senescence and ECM imbalance, offering a transformative strategy for regenerative dermatology and personalized anti-aging approaches.

## Highlights

• SF effectively rejuvenates senescent fibroblasts and restores ECM homeostasis.
• SF suppresses the SASP and enhances cellular antioxidant defense mechanisms.
• SF modulates the ROS–MAPK–AP-1–MMP signaling axis to inhibit collagen degradation.
• SF promotes collagen regeneration via a non-inflammatory pathway, offering a novel regenerative strategy.

## Graphical abstract

**Figure:**
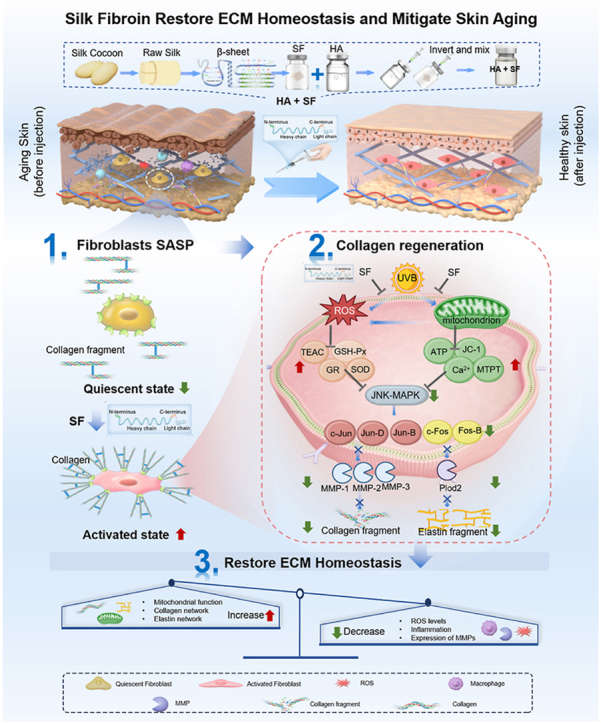

## 1 Introduction

The progressive decline of skin regenerative capacity is a fundamental cause of skin aging. This decline originates from fibroblast senescence, which drives the senescence-associated secretory phenotype (SASP)[1], characterized by enhanced inflammation and reduced collagen synthesis. As a result, mitochondrial dysfunction, oxidative stress, and chronic inflammation occur. SASP further promotes the accumulation of ROS and activation of MMPs[2], accelerating the breakdown of ECM components such as collagen and elastin. The ECM is essential for maintaining dermal architecture, elasticity, and resilience. Its disruption reflects aging progression and exacerbates the loss of tissue regenerative capacity[3]. From a regenerative medicine perspective, restoring fibroblast function and ECM homeostasis is a central strategy for anti-aging interventions.

**Scheme 1:**
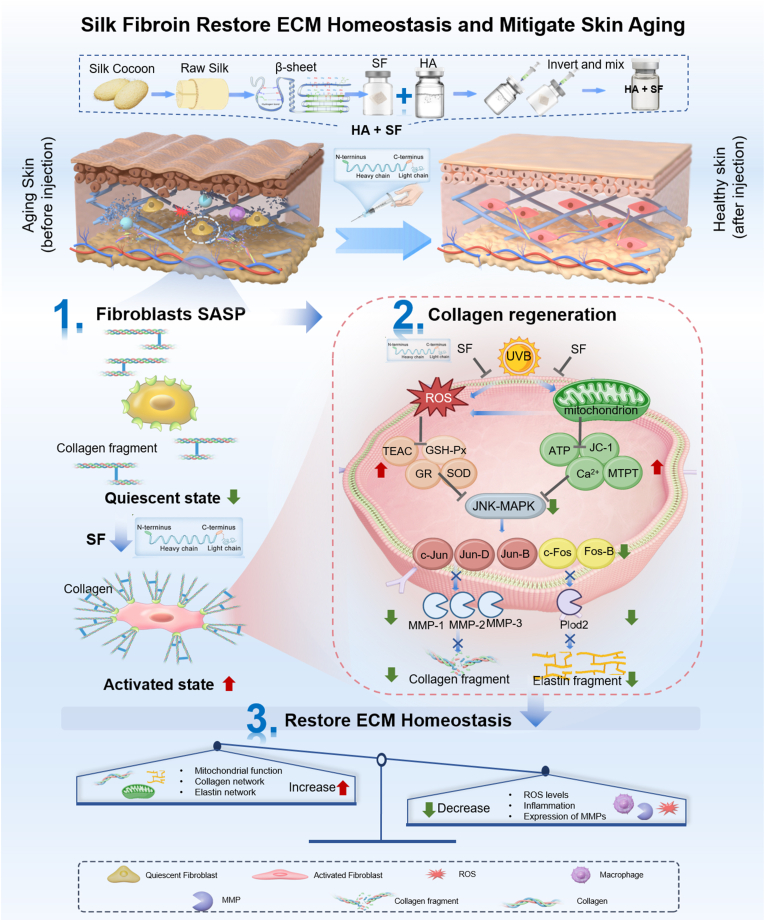
Schematic illustration of the anti-aging effects of SF in restoring ECM homeostasis by modulating fibroblast senescence and promoting collagen network regeneration.

Clinically, dermal injectables are widely employed to counteract soft tissue loss and wrinkles. Hyaluronic acid (HA), collagen, and synthetic polymers are commonly applied. However, these materials primarily target collagen supplementation and cannot modulate ECM homeostasis. HA fillers provide rapid volume restoration but lack regenerative capability[4]. Synthetic polymers such as poly-L-lactic acid (PLLA) and polycaprolactone (PCL) stimulate collagen production through inflammatory foreign-body reactions, which carry risks of fibrosis and nodule formation[5], [6], [7]. Collagen injections offer direct ECM replenishment but suffer from rapid degradation, low bioavailability, and potential immunogenicity[8].These limitations underscore the unmet need for biomaterials that can directly modulate fibroblast senescence and restore ECM homeostasis.

Bioactive peptides (such as signal peptides and collagen peptides) and natural or recombinant proteins hold significant research potential in promoting fibroblast proliferation, migration, and other activities[9], [10], [11]. SF, a natural protein derived from silkworm cocoons, exhibits excellent biocompatibility and a distinctive β-sheet crystal structure, making it suitable for tissue engineering[12], drug delivery[13], and wound healing[14]. As a dermal filler, SF can self-assemble into fibrous scaffolds whose β-sheet conformation resembles certain structural features of the ECM, thereby supporting fibroblast adhesion and proliferation[15], [16]. Our previous study[17] demonstrated that SF significantly enhances dermal fibroblast proliferation and cell adhesion. In vitro studies confirmed that SF-treated fibroblasts exhibited increased type I and III collagen. SF augmented collagen content in the dermis of murine subcutaneous and UVB photoaged skin models. Proteomic analyses further indicated that SF promoted well-organized collagen deposition and ECM reconstruction. Consistent with these findings, other studies have shown that SF-based wound dressings, scaffolds, and microneedles accelerate wound healing[18]. For instance, SF scaffolds improved collagen density and skin elasticity in burn models[19], and SF/HA hydrogels promoted granulation tissue formation and wound closure in diabetic ulcers[20].

SF demonstrates promising potential in ECM remodeling and skin tissue repair. However, its role in regulating fibroblast senescence and ECM homeostasis remains unclear, limiting further clinical translation. Several key scientific questions remain: (1) the signaling pathways by which SF regulates collagen metabolism and whether these differ from the inflammatory mechanisms employed by synthetic materials such as PLLA; and (2) an insufficient understanding of regulating fibroblast senescence and the ECM homeostasis.

To address these gaps, this study focuses on the anti-aging effects of SF through two main approaches: (1) evaluating SF’s ability to promote type I/III collagen and elastin, thereby restoring ECM homeostasis and compared with PLLA; (2) elucidating the underlying mechanisms by which SF improves ECM remodeling, focusing on SASP suppressing, while inhibiting ECM degradation via the MAPK/AP-1/MMP signaling pathway. This non-inflammatory, ECM-focused strategy provides a mechanistic basis for next-generation injectable anti-aging biomaterials. It holds promise for personalized skin regeneration approaches, where the regimen can be tailored to individual ECM and cellular states. Collectively, these results establish SF as a versatile platform for regenerative and precision anti-aging interventions, bridging the gap between conventional fillers and true tissue rejuvenation.

## 2 Methods

### 2.1 Materials

HA was procured from Bloomage Biotechnology Corporation Limited (Shandong, China). SF (average molecular weight: 10–50 kDa) was provided by XianKe Biotechnology Co., Ltd. (Shanghai, China). Lyophilized type III collagen powder for injection (Col III) was obtained from Jinbo Biopharmaceutical Co., Ltd. (Shanxi, China). Injectable HA solution (HA Pro) was purchased from Imeik Technology Development Co., Ltd. (Beijing, China). Injectable PLLA microspheres were supplied by Changchun SinoBiomaterials Co., Ltd. (Jilin, China).

### 2.2 Cells and Animals

L929 cells, ESF-HF-1 cells, and HaCaT cells were obtained from the Cell Resource Center, Chinese Academy of Medical Sciences (Beijing, China). Horse serum (HS), fetal bovine serum (FBS), Dulbecco’s Modified Eagle Medium (DMEM), L-glutamine, penicillin, streptomycin, and 0.25% trypsin were purchased from Thermo Fisher Scientific (Massachusetts, USA).

L929 cells were cultured in DMEM supplemented with 10% (v/v) horse serum, 1% (v/v) L-glutamine, 100 U/mL penicillin, and 100 μg/mL streptomycin. ESF-HF-1 cells were maintained in DMEM containing 10% (v/v) fetal bovine serum, 100 U/mL penicillin, and 100 μg/mL streptomycin. HaCaT cells were cultured in DMEM supplemented with 15% (v/v) fetal bovine serum, 100 U/mL penicillin, and 100 μg/mL streptomycin. All cells were incubated at 37°C in a humidified atmosphere with 5% CO_2_.

Female ICR mice (6–8 weeks old), female BALB/c nude mice (6–8 weeks old), and female adult New Zealand rabbits (1.8–2 kg) were purchased from HFK Bioscience Co., Ltd. (Beijing, China). All animal experiments complied with protocols approved by the Animal Care & Welfare Committee (Nos. 00001560, 00001823, 00001834, and 00002040).

### 2.3 SF’s multifaceted effect on collagen and elastin

SF was dissolved in a complete culture medium to prepare 0.1% and 0.5% solutions. ESF-HF-1 cells were seeded into 96-well plates at 1.5 × 10^4^ cells/mL and co-cultured with different SF concentrations for 7 d. The control group was treated with SF-free DMEM. After culture, the supernatant was collected, and type I collagen, type III collagen, and elastin were measured using ELISA kits (NOVUS, USA). Total RNA was extracted from the cells, and q-PCR was used to analyze the expression of Col I, Col III, and elastin genes.

Non-inflammatory collagen-promoting properties of SF: Co-culturing RAW264.7 and L929 cells to simulate collagen regeneration with inflammatory involvement and culturing L929 cells alone to model regeneration without inflammation. L929 cells were seeded at 8 × 10^3^ cells/mL, and RAW264.7 at 1 × 10^4^ cells/mL in 96-well plates. PLLA, SF, and TGF-β were dissolved in DMEM at 0.5%. TGF-β, purchased from Abcam (Beijing, China), was a positive control. After 7 days, the supernatants were collected, and type I and III collagen levels were measured using ELISA kits (NOVUS, USA).

NF-κB pathway blockade assay: To test whether HA–SF–mediated collagen induction depends on NF-κB/TLR4 signaling, fibroblasts (prepared as described above) were seeded and allowed to adhere overnight. Cells were assigned to the following groups: Control; Control + LPS (positive control, 100 ng·mL^-1^); BAY 11-7082 (NF-κB inhibitor, 5 μg·mL^-1^) + Control; BAY 11-7082 + HA–SF; BAY 11-7082 + SF; and BAY 11-7082 + PLLA. BAY 11-7082 was applied 1 h before the addition of test materials or LPS. For pathway readouts, cells were harvested at 24 h post-treatment for gene expression and immunofluorescence analyses; for ECM outcome measures, supernatants and cell layers were collected at day 5–6 for protein quantification. Specifically, qPCR was performed to quantify transcript levels of IκBα, TLR4, MyD88, and the senescence marker p16. Immunofluorescence staining was used to assess the nuclear localization of phosphorylated p65 (p-p65). Secreted Col I, Col III, MMP-1, and MMP-3 were quantified by ELISA according to the manufacturer’s instructions. All experimental conditions were performed in biological triplicate (n = 3), and each assay included technical replicates.

For the collagen synthesis cycle experiment, L929 cells were seeded at 2,500 cells/well in 96-well plates and co-cultured with SF. The experimental group was treated with a complete medium containing SF, while the control group used a medium without SF. Samples were collected on 2, 5, 8, 12, and 16 d. Col I and Col III expression levels were assessed using Q-PCR and ELISA. The SF-to-CTRL group data ratio was calculated at each time point, eliminating the background effect of the culture medium to determine SF’s net impact on collagen synthesis.

### 2.4 Biocompatibility and activity of HA-SF microinjection formulation

Recombinant SF was extracted from silkworm cocoons (Bombyx mori) as follows: Raw silk was degummed with a 0.02 M Na_2_CO_3_ solution to remove the sericin (60-80°C, 50-80 min). The resulting precipitate was washed with water and dissolved in a 9.3 M LiBr solution. The LiBr was then removed by dialysis (5-15 kDa). Depending on the treatment conditions, SF solutions with different molecular weights were prepared and freeze-dried for long-term preservation under refrigerated conditions.

To prepare the HA-SF formulation, the freeze-dried SF (10-50 kDa) was rapidly re-dissolved in water. Hyaluronic acid (HA) with a molecular weight of 1.5–2.0 MDa was dissolved in water at 4°C, and then mixed with the re-dissolved SF. The mixture was allowed to stand for 1 minute to ensure uniform interaction, while avoiding repeated drawing through a syringe. The formulation was stored at 4°C for further use.

Mechanical properties were determined using a Kinexus Lab+ rotational rheometer (Netzsch, Germany), with frequency sweep measurements performed at a stress of 1% and a frequency range of 0.01–1 Hz. The G' and G'' values were recorded at a frequency of 0.1 Hz. For Fourier transform infrared spectroscopy (FTIR), approximately 2 mg of the freeze-dried hydrogel sample was ground with potassium bromide, pressed at 20 MPa, and analyzed using an iS10 FT-IR spectrometer (Thermo Fisher, USA) over a wavenumber range of 400–4000 cm^-1^. Shear thinning measurements were conducted using a Kinexus Lab+ rotational rheometer with a shear rate ranging from 0.1–100 S^-1^, a Ramp time of 0:00–02:00, and a sample per decade of 10. Mercury porosimetry measurements were conducted using an AutoPore IV 9620 automated mercury porosimeter (Micromeritics, USA). Push force measurement: The sample was filled into a 2.25 mL syringe, a 30G injection needle was installed, a small amount of air at the front end of the syringe was discharged, and then installed on the universal material testing machine (Jinan Zhongzheng ZDW-T100, China), and the testing parameters of the universal material testing machine were set.

Cell compatibility: The HA-SF formulation was tested at its original concentration using Transwell plates to simulate the cellular environment more accurately. L929 cells were seeded at 6,000 cells/well in 24-well transwell plates with 0.2 mL of the formulation, while ESF-HF-1 cells were seeded at 9,000 cells/well in 12-well transwell plates with 0.4 mL of the formulation. Cells were cultured for 6 days in a CO_2_ incubator at 37°C. Cell viability was assessed using CCK-8 at 450 nm (Solarbio, China). Live/Dead staining (Beyotime, China) was performed, with cells treated with 75% ethanol serving as the positive control. Fluorescence microscopy was used for observation. The levels of pro-inflammatory cytokines in the culture supernatant were measured using flow cytometry cytokine detection kits, following the manufacturer’s protocol.

Tissue compatibility: Fifty healthy adult female ICR mice (4–6 weeks old) were individually marked, weighed, and randomly divided into five groups based on implantation periods: 3 d, 1 w, 2 w, 3 w, and 4 w (n=10 per group). The mice were shaved on their backs, and the skin was wiped clean before injection. In a 1 × 1 cm exposed skin area, nine intradermal injection points were marked per mouse, with approximately 0.03 mL of fluid injected per point. The injected skin naturally formed small elevations, which subsided within 1–2 d. Post-injection, the skin reaction was monitored daily for one week using thermal imaging (FLIR, USA) to assess bleeding, necrosis, or sample extrusion. The volume of the raised skin area was recorded as an indicator of sample degradation.

At the end of the implantation period, animals were euthanized, and the injection sites were dissected for pathological examination. The subcutaneous and muscle tissues were inspected for abnormalities and analyzed for hemorrhage, edema, cyst formation, or hyperplasia. Tissue samples (0.5–1.0 cm surrounding the injection site) were collected and fixed in 4% paraformaldehyde for histological analysis. Tissue responses were assessed by fibrous capsule thickness, inflammatory cell infiltration, and any abnormal reactions at the sample-tissue interface.

Fifteen adult female New Zealand rabbits (1.8–2.5 kg) were acclimatized for 2 w and randomly divided into four groups: PBS control group (2 mL/kg), HA-SF formulation (2 mL/kg), commercial HA solution (2 mL/kg), and commercial freeze-dried type III collagen (2 mL/kg). Following restraint, the dorsal fur was shaved, and nine injection sites (0.03 mL/site) were marked within a 1×1 cm area. After the injection, the skin was allowed to return to its natural position. Body temperature and clinical signs were monitored daily. Tissue samples were collected at 7, 14, and 28 d post-injection. Identical histological processing protocols (fixation, paraffin embedding, sectioning, and H&E staining) were applied as in the murine experiments. Histopathological evaluation included measurement of the fibrous capsule thickness, assessment of inflammatory cell infiltration, and analysis of tissue sample interface abnormalities.

To investigate the in vivo degradation profile of SF following dermal administration, a [^3^H]-labeled SF composite solution ([^3^H] composite solution) was injected into the dermis of male and female New Zealand rabbits. Radiolabeled tracing techniques were applied to monitor the metabolic fate of [^3^H]-SF after a single administration. First, plasma, urine, and fecal samples were analyzed using high-performance liquid chromatography coupled with radiometric detection (HPLC–radio) to obtain the corresponding radioactive metabolite profiles. Second, major radioactive metabolites present in plasma, urine, and feces were identified using a combination of HPLC–radioanalysis and mass spectrometry.

In Vitro collagen efficacy test: L929 cells (8×10^3^ cells/mL), ESF-HF-1 cells (1.0×10^4^ cells/mL), and HaCaT cells (2×10^4^ cells/mL) were seeded in 96-well plates. The cells were co-cultured for 7 days with different formulations, while the control group received complete medium. After the culture period, the supernatant was collected, and the levels of type I collagen, type III collagen, and elastin were quantified using ELISA kits. The cells were harvested for total RNA extraction, and q-PCR was performed to analyze Col I, CoI III, Elastin, FLG, AQP3, and Claudin1 gene expression levels.

In vivo efficacy experiment in mice: Skin samples were collected from mice injected with different formulations and analyzed. Masson’s trichrome staining was performed to differentiate collagen fibers (blue) and muscle fibers (red) in the dermis. The stained collagen and muscle fibers were quantified in scanned images. Additionally, polarized light microscopy with Picrosirius Red (PSR) staining was used to distinguish type I collagen (red) and type III collagen (green), followed by quantitative image analysis. Immunofluorescence (IF) staining was further conducted to measure the content of type I and type III collagen in tissue sections.

In Vivo efficacy experiment in New Zealand rabbit: Skin physiological parameters, including moisture content, elasticity, and collagen levels, were measured in New Zealand White rabbits from 0 d to 30 d post-injection using the DermaLab Combo® skin physiology analyzer (Cortex Technology). Measurement protocols and evaluation criteria are detailed in the Supporting Information. New Zealand rabbit efficacy experiment: Skin physiological measurements, including moisture content, elasticity, and collagen levels, will be taken from the New Zealand rabbits 0-30 days after injection using the DermaLab Combo 4 (Cortex) skin physiology analyzer. The principles and indicators of the measurement are detailed in the supporting information.

### 2.5 Transcriptome data analysis based on UVB aging models

Cellular senescence model establishment: ESF-HF-1 cells were seeded at a density of 10,000 cells per well in 6-well plates. Upon reaching 60–80% confluence, the culture medium was discarded, and 0.5 mL of PBS buffer was added to cover the bottom of the plate. The UVB cell irradiator was turned on and allowed to stabilize for 5–10 min. A radiometer was used to mark the UV light intensity (1 mW/cm^2^) at a fixed height and horizontal position. The cell culture plate was placed at the marked position, and the cover was quickly removed. The cells were irradiated for 30 seconds (30 mJ/cm^2^ dose). After irradiation, the plate was promptly removed from the irradiation position, and the PBS buffer was discarded. Complete medium (or medium containing test components) was then added. The cells were then cultured for 2–7 d before subsequent experiments.

Validation of the senescence model: Cell proliferation was assessed using the CCK-8 assay kit, following established protocols to validate the senescence model. Additionally, senescence-associated β-galactosidase staining was performed using a β-galactosidase staining kit (Beyotime, China). The procedure was as follows: The culture medium was removed, and the cells were washed once with PBS. Next, β-galactosidase staining fixative was added, and the cells were fixed at RT for 15 min. After removing the fixative, the cells were washed three times with PBS. Subsequently, 1 mL of staining working solution was added to each well, and the cells were incubated overnight at 37°C on a shaker. Finally, the staining results were observed and recorded under an optical microscope (Biotek, USA).

Transcriptomics analysis: Transcriptomic sequencing was performed by Novogene Co., Ltd (Beijing, China). Differential gene expression between comparison groups was analyzed using DESeq2 software (version 1.20.0). DESeq2 employs a negative binomial distribution model to determine differential expression in RNA-seq data. The Benjamini-Hochberg method was applied to adjust p-values and control the false discovery rate. Genes with an adjusted p-value < 0.05 were classified as differentially expressed.

### 2.6 Investigation of the mechanisms of regulation of skin aging by SF

Mitochondrial dysfunction was evaluated in UVB-induced senescent ESF-HF-1 cells following treatment with different compositions for 6 days, using multiple indicators of mitochondrial function. Mitochondrial calcium (Ca^2+^) levels were measured using the Fura-2 fluorescence probe (Beyotime, China). Fura-2 binds to calcium ions and generates fluorescence, with fluorescence intensity positively correlating with the concentration of mitochondrial Ca^2+^, reflecting mitochondrial metabolic activity and ATP generation capacity. The degree of mitochondrial permeability transition pore (MPTP) opening in senescent cells was assessed using an MPTP assay kit (Beyotime, China), where higher fluorescence intensity indicated better mitochondrial membrane integrity, lower MPTP opening, and stronger mitochondrial function. Mitochondrial membrane potential (ΔΨm) was evaluated using the JC-1 fluorescence probe (Elabscience, China), where healthy mitochondria with high membrane potential formed J-aggregates and emitted red fluorescence. In contrast, a lower membrane potential resulted in JC-1 existing as monomers and emitting green fluorescence. Mitochondrial ATP content was measured using a chemiluminescent ATP assay kit (Elabscience, China) to assess the function of mitochondrial energy metabolism. All assays were performed according to the kit instructions, and fluorescence images were statistically analyzed.

Antioxidant capacity and ROS-related indicators detection: after culturing senescent cells with different compositions, cells were lysed, and proteins were extracted to measure the activities of antioxidant enzymes, including Glutathione Peroxidase (GSH-Px, Mietek, China), Superoxide Dismutase (SOD, Mietek, China), and Glutathione Reductase (GR, DiNing, China) using ELISA kits. The total antioxidant capacity was measured using an ABTS assay kit (Beyotime, China) to assess the antioxidant capacity in New Zealand rabbit tissues and senescent cells. ROS levels were detected in real-time using the DCFH-DA fluorescence probe (Beyotime, China). All experiments were strictly performed according to the kit instructions, and fluorescence images were statistically analyzed.

### 2.7 Validation of SF-regulated skin aging mechanisms in vivo

UVB-Induced skin organoid model: Guangdong BioCell Biotechnology Co., Ltd. established the skin organoid model. The organoid comprised an upper epidermal layer of HaCaT cells and a lower dermal layer of HF-1 fibroblasts. Daily irradiation with UVA and UVB was performed, followed by administration of different formulations (HA, HA Pro, HA-SF, and SF) beneath the medium. The non-irradiated group served as the blank CTRL, while the irradiated untreated group represented the negative CTRL. The positive CTRL group was Vit. C and Vit. E. After 4 days of treatment, samples were collected for analysis.

Skin elasticity was measured using a skin physiology analysis system (Derma Lab Combo 4, Cortex). HE and Masson's staining (provided in SI) and key proteins were assessed by IF, including type I collagen (1:1000, Starter), type III collagen (1:500, Abcam), c-Jun (1:400, Abcam), MMP-1 (1:400, Novus), Jun B (1:50, Abcam), MMP-3 (1:1000, Abcam), JNK (1:500, Abcam), and AP-1 (1:100, Sigma). IF intensity was quantified after imaging.

UVB-Induced nude mouse model: Details of the model establishment are provided in the SI. The animals were randomly assigned to groups before modeling and treatment to minimize potential bias. The skin samples were collected after 4 weeks of treatment. Total RNA was extracted, and tissues were fixed for further analysis. The expression of key genes in the JNK/AP-1/MMP-1 pathway was quantified by q-PCR (primer sequences in SI). IF was performed to evaluate the protein levels of AP-1 (1:500, Sigma) and MMP-1 (1:500, Abcam) in skin tissue.

Experiments on Bama pig photoaged skin were performed by Guangdong Huawei Testing Co., Ltd. Six healthy adult female Bama pigs (≈25 kg) were used, with negative control, positive control, and six treatment groups (HA-SF, HA, HA Pro, Col III, PLLA, SF). The animals were randomly assigned to groups before modeling and treatment to minimize potential bias. Different dorsal regions of the same pig served as repeated injection sites, with each region having eight sites. UV-induced photoaging was established by daily exposure to UVA and UVB for 30 min over 30 days. Subcutaneous injections (0.5 mL per site) were administered twice at 1 d and 3 d after the start of UV exposure. Three pigs were sacrificed at 15 d, and the remaining three at 30 d. Skin samples (1.5 × 1.5 cm) were collected from all injection sites, paraffin-embedded, and processed for histology and molecular analyses. Assessments included H&E staining, Masson’s trichrome, Victoria Blue, immunohistochemistry (elastin, MMP-1), and IF (Col I, Col III, c-Jun, Jun-B, JNK, AP-1).

Collagenase activity measurement: Collagenase activity in the senescent animal model was measured using a Collagenase Activity Assay Kit (colorimetric method, Abcam), which included positive and inhibitor control wells. AP-1 Protein Content Measurement: In addition to qPCR, AP-1 protein content in animal tissues was measured using an AP-1 ELISA kit (Mouse Transcription Factor/Activator Protein 1 ELISA Kit, MyBioSource), with all-trans retinoic acid as the positive control. All experiments followed the kit instructions.

JNK Overactivation and Reversal Assay: To further examine the regulatory role of SF on the MAPK–AP-1 pathway, a JNK overactivation model was established. Cells were pretreated with SF (typically 0.1–0.2%) for 24 h (T = −24 h), followed by incubation with the JNK inhibitor SP600125 (10 μM) for 1 h (T = −1 h). JNK activation was induced by Anisomycin (1 μg/mL) at T = 0. At 60 min, c-Jun nuclear translocation was assessed by immunofluorescence. At 24 h, C-JUN, JUN-D, JUN-B, p16, p21, and AP-1 transcriptional activity were quantified by qPCR. At days 5–6, MMP-1, MMP-3, Col I, and Col III secretion levels were measured by ELISA.

Western Blot (WB): Cells were scraped, collected, and lysed with 100 μL RIPA buffer (Beyotime, China) for 20 min. Samples were transferred to PVDF membranes, blocked with 5% non-fat milk, and incubated overnight at 4°C with primary antibodies: Type I Collagen (1:5000; Starter), Type III Collagen (1:1000; Absin), c-Jun (1:2000; Abcam), MMP-1 (1:1000; Novus), Jun B (1:200; Abcam), MMP-3 (1:2000; Abcam), JNK (1:1000; Abcam), AP-1 (1:200; Sigma), and GAPDH (1:10000; Abcam). Membranes were then incubated with a secondary antibody (1:10000; Abcam) at RT for 2 h. Results were normalized to GAPDH, and grayscale data were analyzed.

Real-time quantitative PCR (q-PCR): Total RNA was extracted from frozen tissues using the RNeasy Mini Kit (Thermo Fisher Scientific, USA). After quantification, 2 μg RNA was reverse-transcribed to cDNA (Superscript III First-Strand Synthesis System, TOYOBO, Japan). qPCR was performed with specific primers **(Table S1)** and SYBR Green (Vazyme, China). Triplicate samples were analyzed using average values.

### 2.8 Statistical analysis

Quantitative data were expressed as mean ± SEM for n ≥ 3. Statistical analysis was performed by one-way or two-way ANOVA, followed by Tukey’s test using GraphPad Prism 9.5.1 for Windows (GraphPad Software, San Diego, California, USA, www.graphpad.com). Statistical significance was defined as ∗p < 0.05, with highly significant differences denoted as ∗∗p < 0.01, ∗∗∗p < 0.001, and ∗∗∗∗p < 0.0001.

## 3 Results and Discussion

### 3.1 SF’s multifaceted effect on ECM

#### 3.1.1 Promotion of collagen and elastin

**Fig. 1:**
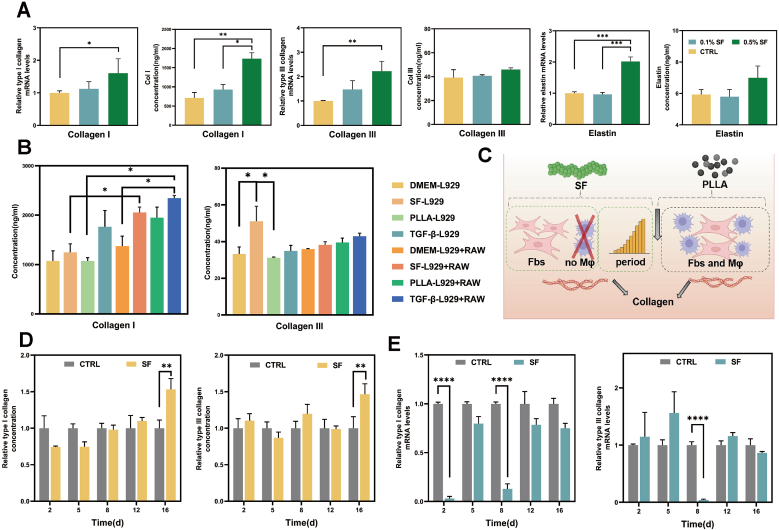
The promoting effect of SF on collagen and elastin regeneration. (A) SF promotes the regeneration of type I and III collagen, as well as elastin (n = 3 trials). (B) Comparison of collagen secretion levels after co-culturing SF and PLLA with cells (n = 3 trials). (C) Schematic diagram of the experimental design showing SF promotes collagen regeneration via non-inflammatory pathways. (D) ELISA analysis identifies SF's promoting effect on collagen secretion (n = 3 trials). (E) Q-PCR analysis identifying the regulation of SF on collagen-related gene expression (n = 3 trials).

Type I collagen, characterized by its thickness and rigidity, forms a network structure supporting the skin. Type III collagen, characterized by its finer filaments and greater elasticity, is dispersed within the type I collagen network, contributing to the skin's smoothness. Previous studies have demonstrated that SF-HA hydrogels improve skin collagen[17]. Further investigation of SF on type I and type III collagen showed that 0.5% SF significantly promotes both types of collagen. The observed effect exhibited a concentration-dependent increase, ranging from 0.1% to 0.5%, accompanied by consistent trends in protein and RNA expression (Fig. 1A).

In addition to its effect on collagen, SF has been shown to influence the production of elastin. Elastin, a protein found in the dermal reticular layer of the skin, forms a fiber network that provides high elasticity and extensibility, enabling the skin to recover its shape after stretching or compression. As elastin degradation occurs due to aging or external factors, such as UV exposure, skin elasticity diminishes, leading to sagging and wrinkles. SF has been demonstrated to promote elastin production at both the RNA and protein levels, with the 0.5% concentration exhibiting the most significant effect in Fig. 1A.

#### 3.1.2 Comparison with existing regenerative materials

Current skin regeneration materials, including PCL and PLLA microspheres, are biodegradable and possess satisfactory mechanical properties, thus making them standard components in facial fillers. However, their collagen-promoting mechanism depends on inflammation. When PLLA microspheres are injected into the skin, they adsorb host proteins and trigger a local inflammatory response[21], [22]. The initial phase of the response is the recruitment of neutrophils, followed by the differentiation of monocytes into macrophages, which engulf the microspheres. After this initial inflammatory response, macrophages and foreign body giant cells release growth and angiogenic factors, stimulating fibroblasts to produce collagen[23].

PLLA was co-cultured with inflammatory cells and fibroblasts to verify this mechanism, successfully replicating its collagen-inducing process. However, when PLLA was cultured with fibroblasts alone, no collagen production was observed, confirming the necessity of inflammatory cells. In contrast, SF alone induced collagen production, differing from the inflammatory-driven mechanism of PLLA (Fig. 1B and C). Molecular markers associated with PLLA-induced inflammation showed no significant increase in SF cultures (Fig. S1).

To further validate that the collagen-promoting effect of HA-SF is independent of inflammatory signaling, we performed an additional set of experiments by selectively inhibiting the NF-κB pathway. Fibroblasts were pretreated with the NF-κB inhibitor BAY 11-7082, followed by exposure to HA-SF, SF alone, or PLLA under identical conditions. LPS stimulation (positive control) markedly increased the expression of TLR4, MyD88, and IκBα, together with enhanced nuclear translocation of phosphorylated p65, confirming the activation of classical inflammatory signaling (Fig. S2). BAY 11-7082 effectively suppressed these responses, validating the inhibitory model.

Under NF-κB blockade, PLLA largely lost its collagen-inducing capacity, consistent with its inflammation-dependent mechanism. In contrast, HA-SF maintained a robust ability to promote collagen production, as evidenced by sustained Col I and Col III secretion at 5–6 d. Notably, HA-SF did not elevate MMP-1 or MMP-3 levels, nor did it increase p-p65 nuclear localization (Fig. S3), indicating the absence of inflammatory activation. Moreover, HA-SF did not upregulate senescence-associated markers such as p16, further supporting its non-inflammatory and non-senescence-inducing profile. Consistently, IF staining of CD86, CD206, and CD68 (Figs. S4–6) showed no increase in pro-inflammatory macrophage polarization, further confirming that HA–SF does not trigger macrophage-mediated inflammatory responses. These results collectively demonstrate that the pro-regenerative activity of HA-SF persists even when the NF-κB pathway is pharmacologically inhibited, highlighting a mechanism that is fundamentally distinct from conventional inflammatory-driven stimulators, such as PLLA.

#### 3.1.3 Adjustment of the collagen production cycle by SF

The collagen production cycle is 14 days in duration[24]. The specific cycle influenced by SF, specifically in relation to RNA transcription and protein secretion, was analyzed. The results revealed two RNA expression peaks, at 5 d and 12 d. Conversely, protein secretion exhibited a gradual accumulation pattern, devoid of a discernible cycle (Fig. 1D and E). The observed consistency between collagen type I and III confirms the hypothesis that SF enhances collagen accumulation in dermal fibroblasts, thereby exceeding the conventional production cycle.

### 3.2 Development and optimization of HA-SF microinjection for anti-aging

#### 3.2.1 Preparation and formulation of HA-SF microinjection

Dermal fillers can generally be categorized into two main types: injectable hydrogels and microneedle-based delivery systems. Injectable hydrogels, studied as volumizing agents for dermal soft tissue deficiencies, serve as physical fillers with effects lasting 1–2 years. In contrast, microneedle injections are a direct delivery method for nutrient solutions into the dermis, resulting in rapid improvement of skin conditions, including dryness and wrinkles. Existing microneedle products can be categorized into two types. One such category comprises HA, which cannot stimulate collagen regeneration. HA is often combined with additives such as vitamin B, carnosine, and amino acids to enhance its bioactivity. The second category directly supplements collagen, with Col III being the most popular due to its unique advantages for the skin. However, the efficacy of these products in addressing skin aging remains to be fully elucidated.

SF for microneedle formulations dissipates rapidly, limiting its efficacy. Additionally, SF lacks the necessary osmotic balance and hydration properties. To enhance its performance, a complementary material was needed. Among various natural polymers, HA was selected for its superior hydration capacity and ECM-like viscosity. The HA-SF microinjection formulation and optimization process have been completed. HA-SF formulation ratio screening is as follows: 1) HA Molecular Weight Screening: With an SF concentration of 0, three different HA molecular weights (<1 MDa, 1-1.8 MDa, >2 MDa) were tested for viscosity and extrusion force. The results indicated that HA with a molecular weight range of 1-1.8 MDa demonstrated the best balance between material operability and stability; thus, this molecular weight range was selected. 2) HA Concentration Screening: Based on the molecular weight selection, different HA concentrations (3–6 mg/mL) were tested for viscosity and extrusion force. A concentration of 5 mg/mL was chosen as the optimal concentration for smooth injection and appropriate viscosity. 3). SF Molecular Weight Screening: Different SF molecular weights (10-50 kDa, 50-100 kDa) were blended with HA solutions, and stability tests were performed at both 4°C and 25°C. Stability, viscosity, and extrusion force at room temperature were considered, and the final selection was made for SF with a molecular weight of 10-50 kDa. 4). SF Concentration Screening: Based on the selected molecular weight, different SF concentrations (1, 3, 5, 7, 9 mg/mL) were tested for the stability, viscosity, and extrusion force of the mixed solution. It was found that concentrations of 5–9 mg/mL exhibited poor stability, while 1–3 mg/mL did not show satisfactory biological activity in cell experiments. After considering osmotic pressure measurement results, a 0.5% HA + 0.5% SF formulation was determined to be optimal.

Rheological characterization of the optimized HA–SF formulation showed a clear shear-thinning profile, indicating good injectability, while G′/G″ and viscosity remained stable at both 4 °C and 25 °C (Figs. S7–S10). The formulation existed as a homogeneous liquid rather than a self-supporting gel; however, porosity analysis **(Table S2)** of the lyophilized blend, together with FTIR and XRD results (Figs. S11–S13), confirmed a uniform HA–SF composite structure without new crystalline transitions.

#### 3.2.2 Safety assessment of HA-SF microinjection

**Fig. 2:**
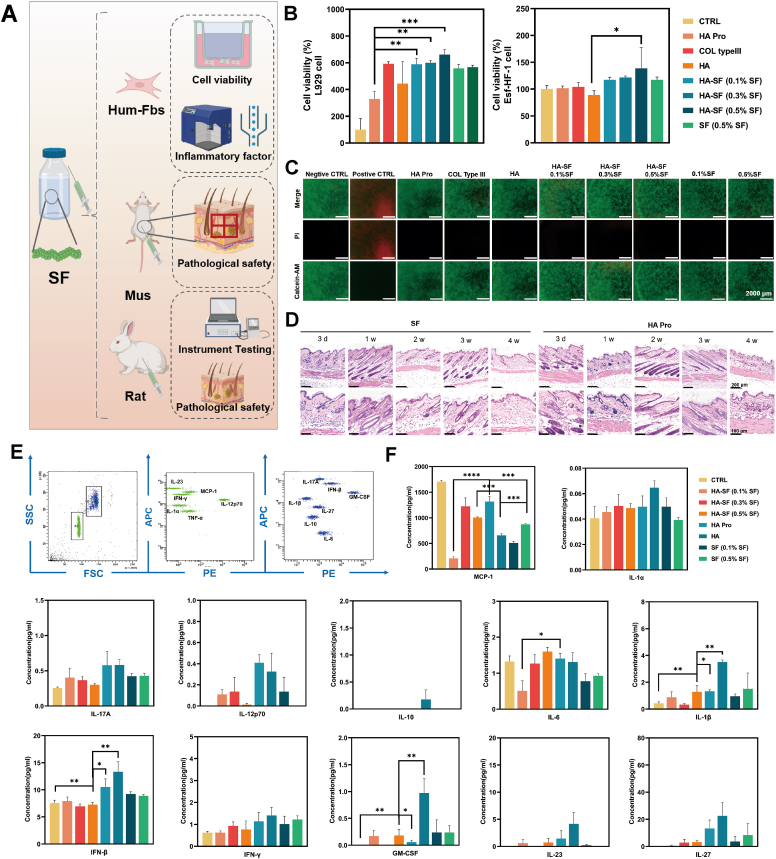
Safety assessment of HA-SF formulation. (A) Schematic representation of the HA-SF safety study. (B) Cell proliferation rates after co-culture of HA-SF with mouse and human fibroblasts (n = 3 trials). (C) Cell viability analysis (Live/Dead fluorescence staining) after co-culture of HA-SF with mouse and human fibroblasts (n = 3 trials). (D) Histopathological analysis of mouse dermis after HA-SF injection (n = 3 mice). (E) Inflammatory cytokine secretion levels (IL-23, IFN-γ, MCP-1, IL-1α, TNF-α, IL-12P70, IL-1β, IL-10, IL-17A, IL-27, IL-6, IFN-β, GM-CSF) after co-culture of HA-SF with human fibroblasts (n = 3 trials).

Biocompatibility is a critical consideration in evaluating medical devices (Fig. 2A). To assess cytocompatibility, permeation assays were conducted using different formulations in the upper chamber, with human and mouse fibroblasts cultured in the lower chamber. The results from cell proliferation assays (Fig. 2B) demonstrated that the HA-SF microinjection system significantly enhanced the proliferation of both mouse and human dermal fibroblasts in a dose-dependent manner (0.1%–0.5%). These findings were further corroborated by live/dead staining, which confirmed high cell viability (Fig. 2C and S14). While SF alone also promoted proliferation, it was to a lesser extent than the HA-SF system. Control groups, including HA Pro, exhibited specific proliferative effects, with collagen III showing the strongest promotion of proliferation.

To evaluate the inflammatory response, we assessed the levels of 13 inflammatory cytokines (IL-23, IFN-γ, MCP-1, IL-1α, TNF-α, IL-12P70, IL-1β, IL-10, IL-17A, IL-27, IL-6, IFN-β, GM-CSF) after co-culture with various formulations (Fig. 2E and F). The results showed that cytokine concentrations remained within the ng or pg range, indicating a low degree of inflammation and a high level of safety. Specifically, the HA-only formulation exhibited the highest levels of cytokines, significantly higher than those of the HA-SF formulation. In contrast, SF alone caused minimal inflammation. Taken together, these findings support the hypothesis that HA-SF has a high safety profile, similar to that of commercial products, while SF demonstrates additional anti-inflammatory properties.

Further validation was conducted using animal models (ICR mice and New Zealand rabbits) to assess tissue compatibility. The 9-point injection technique was used to simulate mechanical injection into the dermis. Throughout the experiment, animals exhibited normal physiological conditions, including stable behavior, appetite, urination, and defecation, with no signs of distress **(Tables S3 and S4)**. No bleeding, necrosis, or material extrusion was observed. At 24 hours post-injection, the injection site showed a slight elevation, which was absorbed by 48 hours.

Histological examination of mouse (Fig. 2D) and rabbit tissues (Fig. S15) revealed intact skin layers at the injection site, with no neutrophil or macrophage infiltration. Biocompatibility scoring **(Tables S5 and S6)**, based on inflammatory cell infiltration and tissue inflammation, showed mild fluctuations at 2 weeks, but remained within a safe range through 4 weeks **(Tables S7 and S8)**. These results are comparable to commercial HA products. In rabbits, HA-SF maintained favorable compatibility scores similar to HA Pro and Col III formulations, all of which were higher than the saline-injected control group (Tables S9 and S10). This suggests that these formulations induce mild external stimulation, similar to foreign materials, which gradually subsides over time, consistent with findings from existing dermal filler studies [25], [26]. The mild inflammatory response observed in HA-SF was well within acceptable limits for water-light formulations.

Moreover, α-SMA immunofluorescence staining revealed that HA-SF did not induce myofibroblast activation, in contrast to the pronounced α-SMA upregulation observed in PLLA-treated tissues. This further confirms the absence of fibrosis-related ECM remodeling (Fig. S16). To evaluate the biodegradation and clearance of HA-SF, a radiolabeled tracing approach was employed. After tritium labeling, the HA-SF solution was injected intradermally, and urine and fecal samples were collected at scheduled intervals to assess the cumulative recovery of radioactive signals. Concurrently, routine blood biochemical analyses were performed to monitor potential systemic effects during the degradation process. The total recovered radioactivity reached approximately 80% by day 10 (Fig. S17), indicating that most of the labeled SF had been metabolized and cleared by this time. These results suggest that HA-SF undergoes a well-defined, biocompatible biodegradation process in vivo, with minimal long-term retention and no detectable abnormalities in blood biochemical parameters **(Tables S11 and S12)**. In summary, comprehensive biocompatibility testing confirms that HA-SF microinjection is safe for clinical applications, exhibiting favorable tissue compatibility, a minimal inflammatory response, and a well-defined biodegradation profile.

#### 3.2.3 Multifunctional effects on skin of HA-SF microinjection

**Fig. 3:**
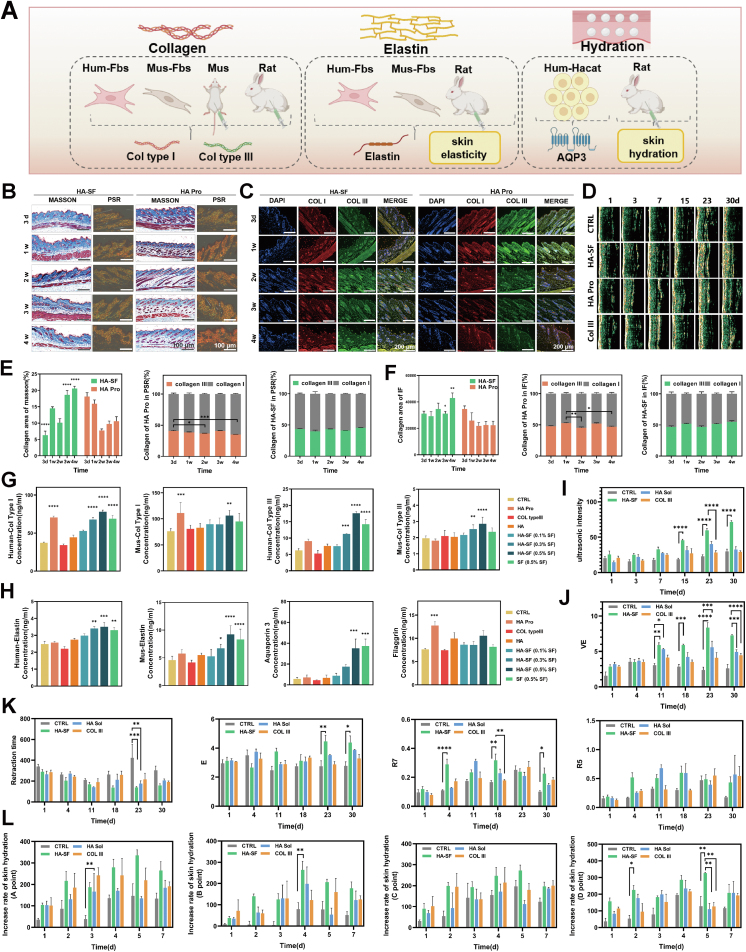
Efficacy study of HA-SF microinjecting formulation. (A) Overview of the experimental design for HA-SF formulation efficacy. (B) MASSON and PSR staining of mouse skin after HA-SF injection (n = 3 mice). (C) IF staining of collagen in mouse skin after HA-SF microinjection (n = 3 mice). (D) Ultrasound measurement of collagen in the New Zealand rabbit after HA-SF microinjection (n = 3 rabbits). (E) Quantitative analysis of type I and type III collagen in PSR staining (n = 3 slices). (F) Quantitative analysis of type I and III collagen in IF staining (n = 3 slices). (G) Type I and type III collagen after co-culture of HA-SF with mouse (n = 5 trials) and human fibroblasts (n = 4 trials). (H) Elastin after co-culture of HA-SF with mouse (n = 5 trials) and human fibroblasts (n = 4 trials), and AQP3 and FLG after co-culture with human epidermal cells (n = 4 trials). (I) Ultrasound measurement of collagen content in rabbit skin (n = 3 trials). (J) Elasticity values (Young's modulus, VE) of rabbit skin (n = 3 trials). (K) Recoil time, elasticity (E), and elasticity curve (R5, R7) of rabbit skin: detailed explanations provided in the supporting information (n = 3 trials). (L) Skin hydration measurement results after HA-SF injection in 4 points of rabbit skin (n = 3 trials).

Beyond meeting the safety requirements of injectable devices, HA-SF formulations also provide multiple bioactive benefits, emphasizing collagen regeneration, hydration, and enhancement of skin elasticity (Fig. 3A). First, we focused on SF's ability to promote collagen regeneration. MASSON staining (Fig. 3B and E) revealed a gradual increase in collagen (blue-stained) from 3 d to 4 w, indicating continuous collagen deposition in the dermis. A comparative analysis of images at 3 d and 1w revealed that HA Pro exhibited a more rapid increase in collagen, as evidenced by the larger blue-stained areas. However, Masson staining is limited in differentiating collagen subtypes, as it evaluates the total collagen content. To distinguish between collagen type I and type III, PSR staining was performed, whereby type III collagen appears yellow-green, and type I collagen appears red. At 3 d, both groups exhibited comparable type I/type III collagen ratios. By 4 w, the HA-SF exhibited a significant increase in type III, while the HA Pro demonstrated a higher proportion of type I collagen. IF quantification (Fig. 3C and F) further validated these observations. The results indicated HA Pro promoted predominantly type I (red fluorescence), resulting in a rapid but transient effect and faster degradation over time. Conversely, HA-SF demonstrated a selective enhancement of type III (green fluorescence), manifesting as a more protracted effect at 3 - 4 w.

During the injection period, we assessed the collagen density in real-time. Collagen has been shown to exhibit higher ultrasound intensity, as indicated by the yellow and white regions, compared to water and blood within the dermis. A substantial increase in collagen density was evident in the HA-SF group on days 23 and 30, exhibiting superior regenerative efficacy (Fig. 3D and I). The results revealed no significant increase at 30 days following the administration of recombinant collagen, suggesting that it has a limited effect or may not be compatible with rabbit models (Fig. S18), which potentially hinders its efficacy. Further validation on human and mouse fibroblasts showed consistent results. HA Pro primarily promoted type I collagen, whereas HA-SF microinjection specifically enhanced type III collagen regeneration, with long-term effects and no risk of fibrosis (Fig. 3G).

In addition to promoting collagen, SF has been shown to enhance skin elasticity and hydration. Skin elasticity is closely related to elastin, which provides resilience in the dermis. In dermal fibroblast models, SF promoted elastin concentration-dependent, with the optimal effect at 0.5% (Fig. 3H). The promotion of elastin by HA Pro is attributable to its amino acid components, which facilitate collagen regeneration. In New Zealand rabbits, real-time skin elasticity measurements showed HA-SF injections improved various skin elasticity indices at 23 d, particularly viscoelasticity (VE) (Fig. 3J, K, and S19). HA Pro also demonstrated satisfactory efficacy, consistent with the results of cell experiments. The Col III exhibited the least improvement, suggesting that collagen supplementation alone cannot enhance skin elasticity.

Skin hydration is associated with epidermal barrier function. Utilizing an epidermal barrier model, alterations in water channel proteins (AQP3) and barrier proteins (FLG, LOR, Claudin1) were measured to evaluate the hydration effects. The results demonstrated that both HA Pro and HA-SF promoted FLG; however, HA-SF also increased AQP3, thereby enhancing water transport in the epidermis (Fig. 3H and S19). In New Zealand rabbits, the HA-SF and HA Pro groups exhibited improved skin hydration, with HA-SF showing superior hydration effects (Fig. 3L).

The HA-SF microinjection demonstrated significant advantages in collagen regeneration, hydration, and elasticity. The promotion of type III collagen regeneration with no risk of fibrosis, the enhancement of elastin for improved elasticity, and enhanced skin hydration through modulation of AQP3 and FLG were all observed.

### 3.3 Mechanism of SF in the regulation of skin aging

#### 3.3.1 Model Construction and Transcriptomic Analysis

The external manifestations of skin aging include skin laxity and wrinkles, both of which are associated with ECM disruption. (1). Sagging: The loss of collagen, a protein that provides structure and elasticity to the skin, leads to a reduction in dermal thickness, causing the skin to lose its support and resulting in collapse and loosening. (2). Wrinkles: A decrease in elastin and moisture loss reduces skin elasticity, leading to the deepening of wrinkles, particularly as dynamic wrinkles transition into static ones. Multiple internal factors cause these changes. For example, fibroblasts age, reducing their ability to synthesize collagen and elastin, disrupting the ECM's stability. The accumulation of ROS accelerates cell damage, activating MMPs and exacerbating collagen degradation. Chronic inflammation, or inflammation, as defined by the presence of pro-inflammatory factors such as IL-6 and TNF-α, further exacerbates the processes above by driving the destruction of the ECM and increasing ROS accumulation. The interplay of these factors culminates in the manifestation of various cutaneous manifestations, including skin laxity, deepened wrinkles, diminished elasticity, and accelerated moisture loss, thereby expediting the skin aging process.

**Fig. 4:**
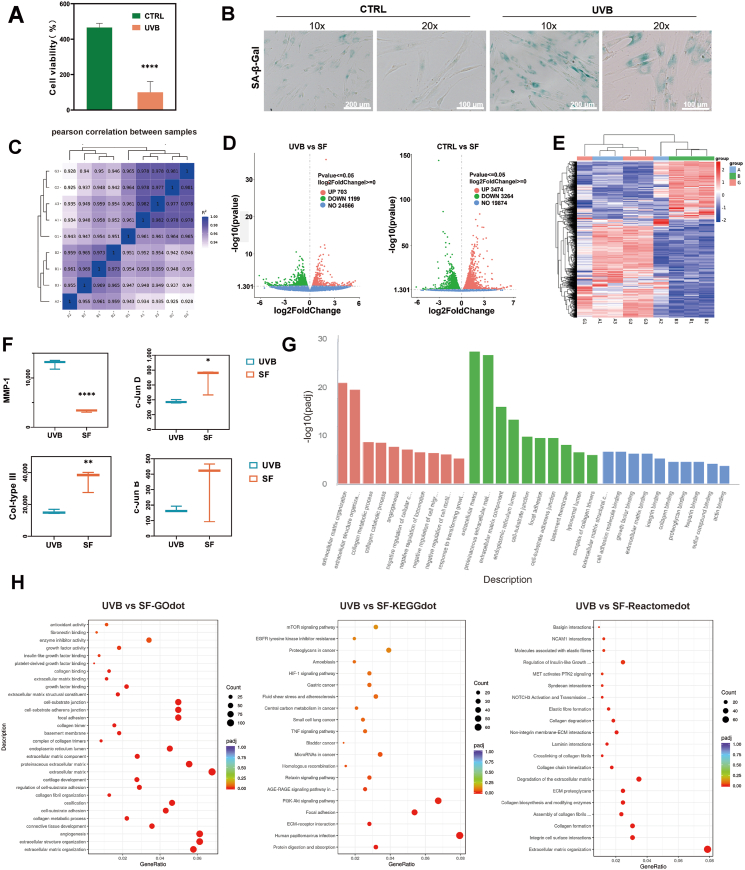
Establishment of UVB-induced Skin Aging Model and Transcriptomic Data Analysis. (A) Characteristics of UVB-induced cellular senescence model: Slower cell proliferation (n = 6 trials). (B) Characteristics of UVB-induced cellular senescence model: Increased β-galactosidase staining (n = 3 trials). (C) Correlation analysis of transcriptomic data among different groups (n = 3 trials). (D) Volcano plots of differentially expressed genes between the UVB and SF treatment groups, and between the CTRL and SF groups (n = 3 trials). (E) Clustering heatmap of differentially expressed genes between the UVB and SF groups (n = 3 trials). (F) Boxplot of differential gene expression between the UVB and SF groups (n = 3 trials). (G) GO enrichment analysis of differential genes between the UVB group and SF group, covering molecular function (MF), cellular component (CC), and biological process (BP). (H) GO enrichment analysis, KEGG pathway enrichment analysis, and Reactome pathway enrichment analysis results from differential genes between the UVB and SF groups.

Most aging models are UVB-induced photoaging models, which simulate skin aging[27]. Following UVB irradiation, a decline in cell proliferation is observed, accompanied by a decrease in cell numbers, an enlargement of cell nuclei, and the secretion of aging-specific enzymes (Fig. 4A). For instance, the use of β-galactosidase staining facilitates the identification of senescent cells, with increased blue staining signifying a greater number of aging cells. The UVB-treated groups exhibited substantial disparities compared to the untreated CTRL group, thereby validating the efficacy of the established model (Fig. 4B).

After co-culturing senescent cells with SF microinjection, transcriptomic analysis was performed, and proteomic analysis was conducted to explore further the underlying mechanism of the cellular behavior properties of SF microinjection. The correlation between samples confirmed a strong correlation within each group, providing a reliable basis for subsequent data analysis (Fig. 4C). In the UVB vs SF group, 26,468 genes were identified. The clustering heatmap displayed the differential genes between the groups. The volcano map in Fig.4D and E showed 1,902 differentially expressed genes, including 703 up-regulated and 1,199 down-regulated genes, marked in red and green, respectively. In the CTRL vs SF group, 26,612 genes were identified, and 6,738 differentially expressed genes were found, including 3,474 upregulated and 3,264 downregulated genes. Among them, the first significant enrichment in UVB vs SF was MMP-1, a key enzyme involved in collagen degradation. This suggests that the mechanism can be explored from the perspective of SF and collagen degradation via MMP-1. Additionally, COL3 was enriched, which is consistent with the previous experimental results (Fig. 4F).

Of note, among the 10 most significantly enriched terms in each category of Gene Ontology (GO) enrichment analysis in UVB vs SF, terms such as extracellular matrix organization, extracellular structure organization, collagen metabolic process, collagen fibril organization, cell-substrate adhesion, and reactive oxygen species metabolic process in the biological process (BP) category, extracellular matrix structural constituent, collagen binding, growth factor binding, fibronectin binding in the molecular function (MF) category, and extracellular matrix, proteinaceous extracellular matrix, extracellular matrix component, collagen trimer in the cellular component (CC) category suggested the collagen regeneration effect and cell proliferation of SF on the injected skin (Fig. 4G and H). The GO enrichment analysis of CTRL vs SF (Fig. S20), including terms such as extracellular matrix organization, extracellular structure organization, positive regulation of cell-substrate adhesion, and regulation of cell-substrate adhesion in the biological process (BP) category, cell-substrate adherens junction, focal adhesion, extracellular matrix component, proteinaceous extracellular matrix in the cellular component (CC) category, cadherin binding, DNA-dependent ATPase activity, extracellular matrix binding, SMAD binding in the molecular function (MF) category, showed more enrichment in ECM and cell adhesion physiological processes. This did not further enrich the collagen-related pathways, further proving the amplification effect of the UVB model on collagen-related mechanisms.

We performed KEGG enrichment analysis to evaluate further the pathways affected by SF (Fig. 4H and S21). In UVB vs SF, proteins were enriched in "Protein digestion and absorption," "ECM-receptor interaction," "Focal adhesion," "NF-kappa-B signaling pathway," and "MAPK signaling pathway." While the GO analysis focused more on molecular biological processes, the KEGG analysis was centered on specific mechanisms, refining the research direction for the mechanism study. Furthermore, using the Reactome database (Fig. 4H and S22), which includes various reactions and biological pathways of human and other model organisms, we found that in UVB vs SF, proteins were enriched in "Extracellular matrix organization," "Collagen formation," "Assembly of collagen fibrils and other multimeric structures," "ECM proteoglycans," and "Degradation of the extracellular matrix," further validating the current effects of SF on skin collagen.

#### 3.3.2 SF-Mediated Modulation of SASP in Senescent Fibroblasts

**Fig. 5:**
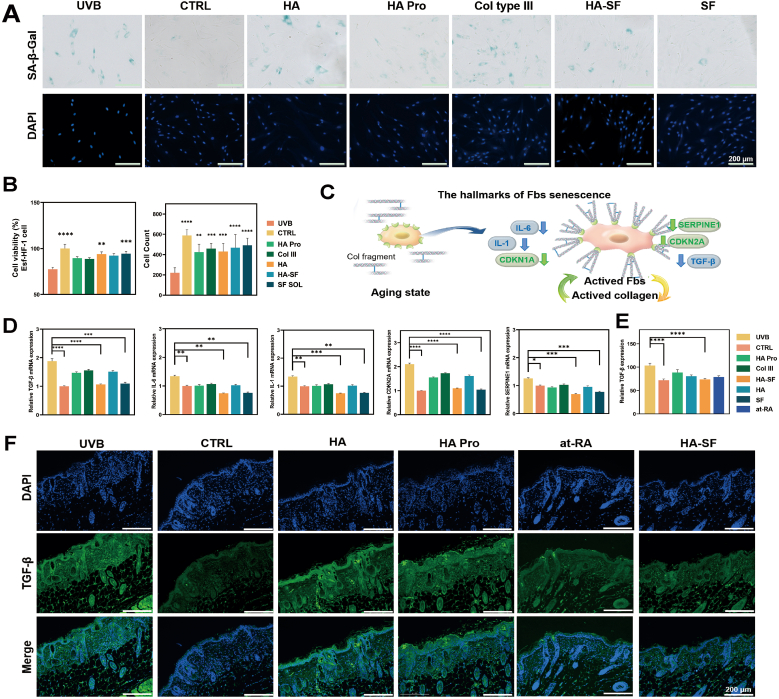
Mechanistic Study of SASP in Senescent Fibroblasts by SF. (A) β-galactosidase staining results of senescent cells in different treatment groups (n = 3 trials). (B) Proliferation rate (n = 6 trials) and cell count (n = 4 trials) results of senescent cells in different treatment groups. (C) Schematic diagram of two states of fibroblasts (activated state vs. senescent state). (D) In senescent fibroblasts, expression levels of specific inflammatory factors, TGF-β, IL-6, IL-1, CDKN2A, and SERPINE1 (n = 3 trials). (E) Quantitative statistical results of TGF-β expression levels in senescent animal models (n = 3 mice). (F) Immunohistochemistry or IF images of TGF-β expression in senescent animal models (n = 3 slices).

Skin aging is closely associated with ECM disorganization, which leads to weakened structural support, impaired nutrient exchange, a decline in fibroblast numbers, and a progressively oxidative and pro-inflammatory microenvironment (Fig. 5A and 5B)[28]. A pivotal event in this process is the activation of the SASP[29]. Activated fibroblasts typically exhibit an elongated spindle morphology, maintained by ECM-derived mechanical cues. Upon ECM disruption, fibroblasts adopt a rounded, quiescent phenotype characterized by reduced activity and elevated SASP expression (Fig. 5C)[29], [30]. The early SASP phase is characterized by inflammatory signals that recruit immune cells, whereas later stages involve the upregulation of MMPs and TIMPs, contributing to ECM degradation and chronic inflammation [31].

SF has demonstrated potential in reversing fibroblast senescence. In UVB-induced aging cell models, SF significantly promoted fibroblast rapid proliferation within 2–3 d, as shown by increased cell counts and CCK-8 assay results. β-galactosidase staining revealed a marked reduction in senescent cells following HA-SF microinjection, indicating a rejuvenated cell population (Fig. 5A and B). Beyond proliferation, SF suppressed key SASP factors, including IL-6, p21 (CDKN1A), and p16 (CDKN2A), as well as SERPINE1, thereby alleviating the pro-inflammatory state and restoring fibroblast functional activity in vitro (Fig. 5D) and in vivo (Fig. 5E and F). Mechanistically, this may involve the inhibition of NF-κB and MAPK signaling, coupled with the regulation of cell cycle checkpoints, which supports the transition of senescent cells toward an activated phenotype capable of ECM repair.

Compared to HA, HA Pro, and Col III, which showed limited ability to mitigate SASP or restore fibroblast activity, the SF-based formulation exhibited superior efficacy across all evaluations. HA-SF microinjection markedly improved fibroblast proliferation and reversed cellular senescence (Fig. 5A and B), while also significantly reducing senescence markers, promoted collagen synthesis, and restored a spindle-like fibroblast morphology indicative of an active state (Fig. 5D). These findings confirm SF’s dual action: not only stimulating fibroblast proliferation but also reversing SASP-induced dysfunction, thereby blocking the pathway of cellular aging and ECM degradation. This highlights the therapeutic potential of SF in restoring a regenerative fibroblast phenotype, which is essential for long-term dermal homeostasis.

#### 3.3.3 SF Remodeling collagen networks by MAPK-AP-1-MMP pathway

**Fig. 6:**
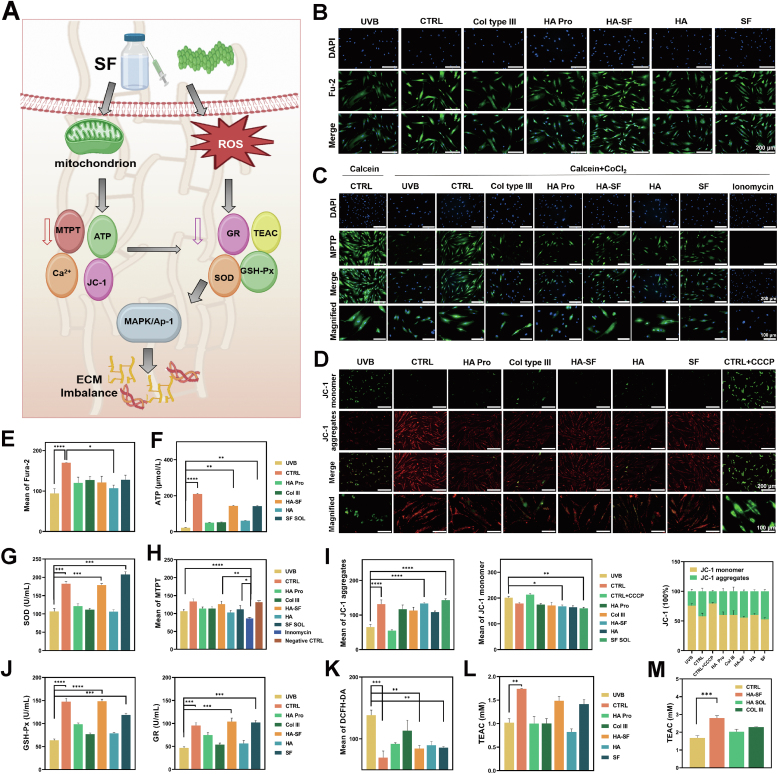
Mitochondrial Regulation and ROS Suppression by SF of collagen regeneration. (A) Schematic representation of the upstream mechanism by which SF improves ECM dysfunction. (B) Mitochondrial Ca^2+^ fluorescence staining images (n = 4 trials). (C) Mitochondrial permeability transition pore (MPTP) fluorescence staining images (n = 4 trials). (D) Mitochondrial membrane potential (JC-1) fluorescence staining images (n = 4 trials). (E) Quantitative statistical results of mitochondrial Ca^2+^ fluorescence staining (n = 4 slices). (F) Quantitative statistical results of mitochondrial ATP content (n = 3 trials). (G) Superoxide dismutase (SOD) content in senescent cells in different treatment groups (n = 3 trials). (H) Quantitative statistical results of mitochondrial MPTP fluorescence staining (n = 4 slices). (I) Quantitative statistical results of mitochondrial JC-1 fluorescence staining (n = 4 slices). (J) Glutathione (GSH) and glutathione reductase (GR) content in senescent cells in different treatment groups (n = 3 trials). (K) Quantitative statistical results of ROS fluorescence staining (n = 3 trials). (L) ABTS antioxidant capacity assay results in different treatment groups (n = 3 trials). (M) ABTS antioxidant capacity assay results in different tissues (n = 3 trials).

Through transcriptomic differential gene analysis and extensive PCR validation, the top-ranked MMP confirmed that the mechanism involved the MAPK-AP-1-MMP pathway. Among the aging pathways, the core driver is ROS, which plays a pivotal role. Senescent cells are characterized by heightened energy-producing oxidative metabolism that surpasses that of proliferating cells, which sustains essential ATP-consuming proteostress pathways vital for survival. The primary source of ROS is mitochondrial dysfunction-induced oxidative phosphorylation, with additional contributions from external environmental factors, such as UV radiation, pollutants, and smoking [32]. Accumulating excessive ROS results in oxidative stress, damaging skin cells and the ECM, consequently accelerating skin aging (Fig. 6A).

SF substantially impacts the mitigation of mitochondrial dysfunction, as evidenced by multiple markers related to mitochondria. Firstly, after SF treatment, the maintenance of calcium ion homeostasis is evidence of SF's potential in regulating mitochondrial homeostasis, preventing Ca^2+^ overload-induced mitochondrial damage and apoptosis (Fig. 6B and E). The degree of mitochondrial permeability transition pore (MPTP) opening is significantly reduced, indicating that SF inhibits the abnormal opening of MPTP, maintains mitochondrial membrane stability, and prevents premature activation of apoptotic signals (Fig. 6C and H). Secondly, JC-1 fluorescence staining (Fig. 6D and I) demonstrates that SF contributes to maintaining a high mitochondrial membrane potential (ΔΨm), thereby preventing mitochondrial depolarization and providing additional evidence for its protective effect on mitochondrial integrity. Additionally, the restoration of ATP levels (Fig. 6F) indicates that SF enhances mitochondrial energy metabolism and improves oxidative phosphorylation efficiency, thus maintaining normal cellular function.

Based on this, the evaluation of the HA-SF microinjection formulation's effects was conducted. The results showed this formulation exhibited analogous improvement trends in all mitochondrial function-related indicators, thereby indicating the HA-SF microinjection also has the potential to repair mitochondrial function and reduce oxidative stress. However, the HA alone did not significantly improve mitochondrial function, suggesting that HA may primarily serve an auxiliary role in this process. Subsequently, a comparative analysis was conducted on the effects of HA Pro and Col III. The results indicated that neither of these two products exhibited a significant improvement in mitochondrial function, as measured by MPTP opening, ATP levels (Fig. 6F), mitochondrial membrane potential (JC-1) (Fig. 6D and I), or calcium ion homeostasis. This further emphasizes the unique advantages of SF and its composite formulations in addressing mitochondrial dysfunction.

The excessive accumulation of ROS is a primary driving factor in skin aging, inducing oxidative damage, inflammatory responses, and ECM degradation. We measured the activity of three key antioxidant enzymes: superoxide dismutase (SOD) (Fig. 6G), which catalyzes the dismutation of superoxide anion (O_2_^-^) to hydrogen peroxide (H_2_O_2_); and glutathione peroxidase (GPx), which further reduces H_2_O_2_ to water. Additionally, we examined glutathione reductase (GR), which plays a pivotal role in maintaining the balance of intracellular glutathione (GSH) and oxidized glutathione (GSSG), thereby protecting cells from oxidative damage (Fig. 6J). To comprehensively assess the effect of SF on reducing ROS levels, we employed fluorescent probe staining to directly measure intracellular ROS content, reflecting the cellular oxidative stress levels (Fig. 6K and S23). Total antioxidant capacity was evaluated using the ABTS method to assess the overall antioxidant ability of the cells (Fig. 6L).

It was observed that the ROS levels were significantly lower in the SF group compared to those of the senescent cells in the CTRL group. At the same time, the activities of SOD, GPx, and GR were significantly increased, along with elevated ABTS values. These findings suggest that the antioxidant capacity of cells is enhanced by SF, leading to reduced damage caused by oxidative stress. The HA-SF microinjection demonstrated a substantial reduction in intracellular ROS levels, indicating that the antioxidant capacity is amplified through the synergistic effect of HA and SF (Fig. 6K and S23). In contrast, no significant antioxidant effects were observed in the HA-only group, implying that only HA makes a marginal contribution to ROS clearance. HA Pro achieved a reduction in ROS, whereas the Col III group showed no significant effects on any antioxidant indicators. Further investigation in UVB-induced skin aging animal models revealed that the HA-SF microinjection had significantly higher total antioxidant capacity than the UVB damage group (Fig. 6M). This finding confirmed the role of SF in inhibiting ROS accumulation and improving antioxidant capacity, providing experimental evidence for its application in skin aging interventions.

**Fig. 7:**
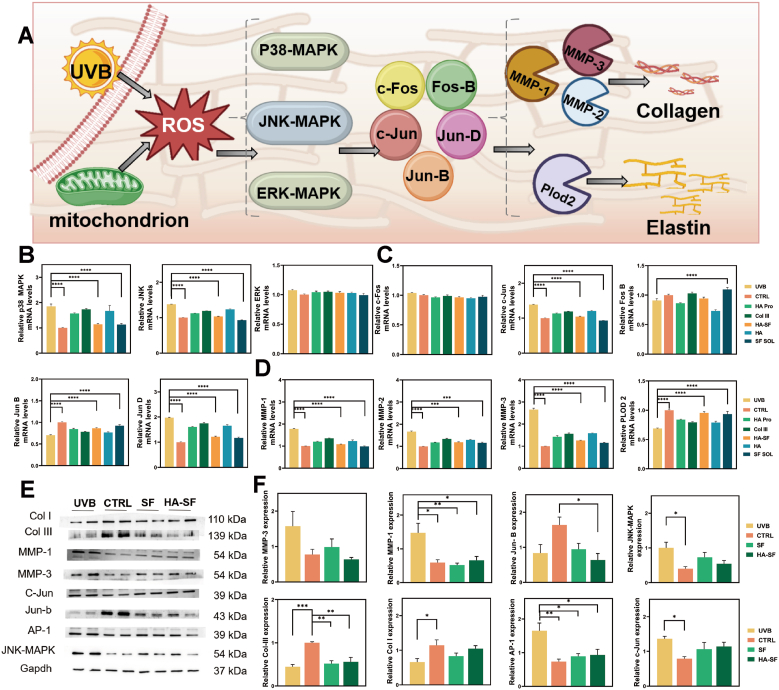
Mechanism of remodeling collagen and elastin networks by SF. (A) Schematic diagram of the mechanism by which SF improves ECM dysfunction. (B) PCR validation of the expression of MAPK in the MAPK-AP-1-MMP pathway (n = 3 trials). (C) PCR validation of the expression of AP-1 in the MAPK-AP-1-MMP pathway (n = 3 trials). (D) PCR validation of the expression of MMP in the MAPK-AP-1-MMP pathway (n = 3 trials). (E) WB validation of the expression of key proteins in the MAPK-AP-1-MMP pathway (n = 4 trials). (F) Quantitative analysis of the grayscale values of WB results (n = 4 trials).

The MAPK pathway is one of the signaling pathways that mediate cellular responses to external stress, especially in skin aging. The activation of the MAPK signaling pathway promotes the expression of MMPs, leading to accelerated collagen degradation. In this study, the regulatory mechanism of SF in the MAPK pathway was evaluated systematically (Fig. 7A).

Q-PCR was used to assess the members of the MAPK protein. The MAPK pathway comprises three major branches: p38, JNK, and ERK, each associated with distinct functions [33]. Among these, P38 strongly correlates with cellular stress and inflammation, JNK is implicated in stress-induced apoptosis, and ERK is primarily responsible for cell proliferation and survival. It was observed that the mRNA levels of JNK and p38 were significantly downregulated after SF treatment. In contrast, ERK remained unchanged (Fig. 7B). The results indicate that SF effectively suppresses the ROS-induced overactivation of the MAPK pathway during skin aging. Further validation by WB and IF analysis revealed that JNK protein expression was more strongly inhibited by SF, as evidenced by a marked reduction in the JNK band intensity (Figs. S24 and S25). These results suggest that the anti-aging effects of SF were mediated predominantly through the inhibition of the JNK pathway.

AP-1 (Activator Protein-1) is a downstream transcription factor in the MAPK pathway. The activation of AP-1 plays a critical role in regulating aging-related genes, particularly in MMPs. AP-1 consists of c-Jun and c-Fos subfamily members, with c-Jun implicated in cellular senescence, inflammation, and collagen degradation. The C-Fos family plays a pivotal role in cell proliferation and tumor formation[33]. Q-PCR results showed that the expression of c-Jun was significantly reduced following SF treatment. In contrast, c-Fos expression was largely unaffected (Fig. 7C). SF has a more pronounced inhibitory effect on c-Jun, further confirming that its regulation of aging-related genes occurs primarily through the MAPK-c-Jun-AP-1.

As downstream effectors of the MAPK pathway, MMPs have been identified as crucial mediators of skin aging. In this process, collagen is first cleaved into large fragments by MMP-1, and then further degraded by MMP-2, MMP-3, MMP-5, and other proteolytic enzymes [34]. Excessive activation of MMP-1 is a significant cause of collagen loss in skin aging. Q-PCR analysis revealed that MMP-1 expression was significantly suppressed by SF, along with reductions in MMP-2 and MMP-3 (Fig. 7D).

Finally, WB experiments were conducted to validate the expression changes of key MAPK pathway proteins conclusively. Results demonstrated a significant reduction in JNK expression in SF-treated groups, corroborating the q-PCR data. Furthermore, SF treatment markedly inhibited the expression of MMP-1 and MMP-3, thereby preventing collagen degradation (Fig. 7E and S25). This confirms that SF suppresses MAPK activation through the JNK-c-Jun-AP-1 signaling pathway, modulating MMP expression (Fig. 7F). Collectively, these results provide compelling evidence for the efficacy of SF in collagen and elastin networks associated with skin aging.

### 3.4 Validation of ECM Homeostasis in SF-mediated anti-skin aging

These findings suggested that SF could restore ECM homeostasis in aged skin. Three models were employed to investigate its role in vivo: a UVB-induced skin organoid model, a photoaged nude mouse model, and a photoaged Bama pig model.

**Fig. 8:**
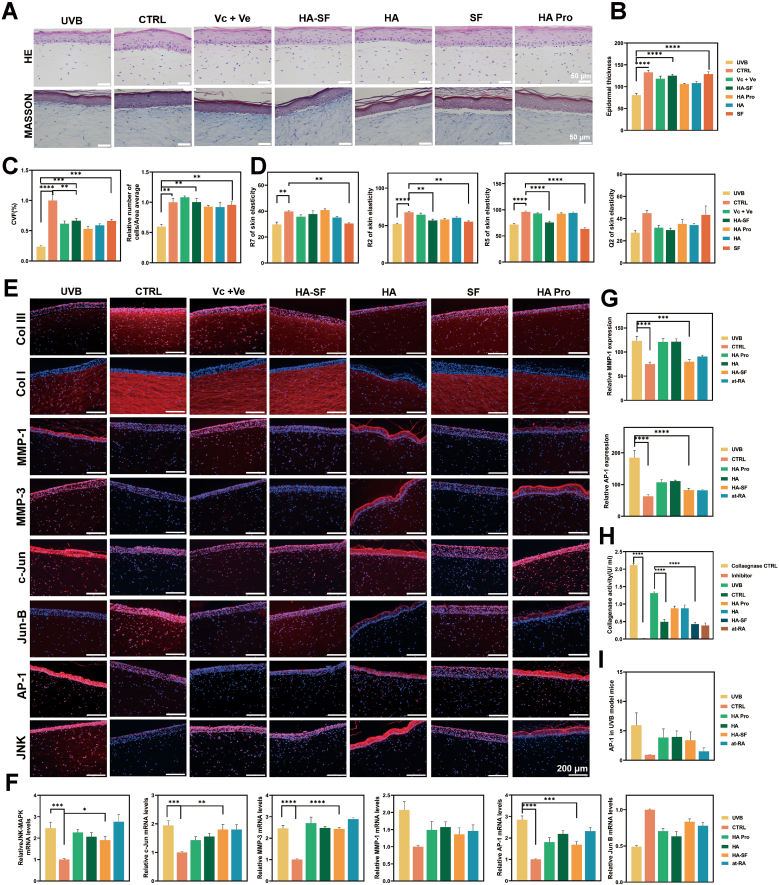
Validation of ECM Homeostasis in SF-mediated anti-skin aging. (A) H&E and Masson’s trichrome staining of 3D organoid senescence model post-treatment (n = 3 trials). (B) Epidermal thickness analysis in H&E-stained 3D organoids (n = 3 trials). (C) Dermal collagen fraction and fibroblast density (cells/area) in Masson’s trichrome-stained 3D organoids (n = 3 trials). (D) Skin elasticity coefficient analysis in 3D organoids (n = 3 trials). (E) MMP-1/AP-1/JNK pathway validation in 3D organoids (n = 3 trials). (F) Quantitative IF staining of AP-1 and MMP-1 in photoaged nude mouse skin (n = 3 slices). (G) Collagenase activity in photoaged nude mouse skin (n = 6 mice). (H) AP-1 protein expression in photoaged nude mouse skin (n = 6 mice). (I) The mRNA levels of MMP-1/AP-1/JNK pathway in photoaged nude mouse skin (n = 3 trials).

In the UVB organoid model, pathological photoaging changes were observed (Fig. 8A and B), including hyperkeratosis, reduced epidermal thickness, and decreased dermal fibroblast density. Masson’s trichrome staining (Fig. 8C) revealed a 72.7% reduction in collagen fiber density after UVB exposure. Microinjection of SF and HA-SF significantly improved epidermal morphology, restored epidermal thickness, and enhanced dermal ECM, accompanied by increased fibroblast proliferation and a higher collagen fraction. SF also enhanced skin biomechanical recovery, as multiple elasticity parameters (R2, R5, R7) approached healthy skin levels after treatment (Fig. 8D and S26). In addition, MMP-1, MMP-3, c-Jun, AP-1, and JNK proteins in the MMP-1/AP-1/MAPK pathway were downregulated following SF treatment (Fig. 8E and S27), whereas Col I, Col III, and Jun-B were significantly upregulated. Notably, AP-1 and MMP-1 exhibited the most prominent changes, suggesting that they play a key role as regulatory targets.

In the UVB-induced photoaged nude mouse model, the HA-SF hydrogel modulated the expression of MAPK-related genes and proteins. Compared with the UVB group, MAPK signaling factors were downregulated in the HA-SF group, indicating effective regulation of this pathway (Fig. 8F). UVB irradiation led to significant upregulation of MMP-1 (Fig. 8G and S28) and AP-1 (Fig. 8G and S29), while SF treatment suppressed their expression to near-normal levels, effectively inhibiting UVB-induced collagen degradation. The positive control, at-RA, also reduced MMP-1 and AP-1 expression; however, its effect was weaker, with a limited impact on the MAPK pathway. Further quantitative analysis confirmed that collagenase activity was significantly lower in the SF group compared with the UVB group (Fig. 8H). ELISA results also validated effective downregulation of AP-1 by SF (Fig. 8I). In contrast, Col III and HA Pro failed to regulate this signaling pathway and did not improve ECM homeostasis.

To further validate the involvement of the MAPK–AP-1 pathway, a JNK-overactivation model was established. Anisomycin markedly enhanced c-Jun nuclear translocation and increased AP-1 activity, leading to elevated MMP-1 and MMP-3, and reduced COL I/III, thereby reproducing a stress-induced aging phenotype. Notably, SF effectively reversed these alterations. Even under sustained JNK activation, SF suppressed c-Jun activation, reduced AP-1–driven MMP expression, and restored collagen synthesis (Figs. S30 and S31).

**Fig. 9:**
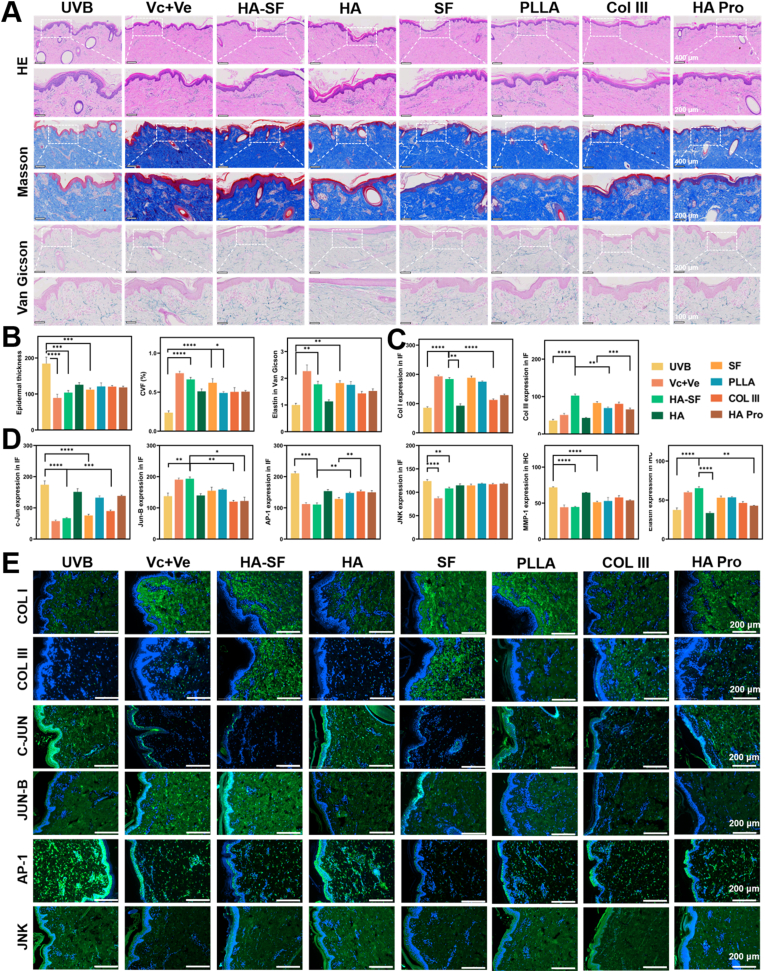
Assessment of ECM remodeling in the Bama pig model. (A) H&E, Masson’s trichrome, and Victoria blue staining of skin sections from the Bama pig senescence model after treatment (n = 6 pigs). (B) Quantification of epidermal thickness, dermal collagen fraction, and elastin content in Fig.9A (n = 3 slices). (C) Quantitative IF staining of COL I and Col III in photoaged pig skin sections (n = 3 slices). (D) Quantitative IF analysis of the MMP–AP-1–MAPK pathway in Fig.9E (n = 3 slices). (E) The MMP–AP-1–MAPK pathway was validated in photoaged pig skin sections at 30 d (n = 3 pigs).

In the photoaged Bama pig model, 30 days of UVB irradiation resulted in hardened and roughened skin **(Table S13)**. Histological analysis revealed increased epidermal thickness and a reduction in collagen content. Following injection with HA-SF or SF, significant improvements were observed in both epidermal structure and ECM composition, particularly in collagen and elastin levels (Fig. 9A and B). Molecular assays further confirmed that SF significantly enhanced ECM content, as evidenced by increased expression of collagen I and III (Fig. 9C and E). Additionally, SF regulated the MMP-1/AP-1/MAPK signaling pathway, promoting the restoration of ECM homeostasis and inhibiting ECM degradation (Fig. 9D, E, and S32). In contrast, while PLLA increased collagen content, it failed to suppress the signaling pathways responsible for collagen degradation, highlighting the superior efficacy of SF and HA-SF in ECM remodeling.

SF alleviated the SASP phenotype of senescent fibroblasts and attenuated collagen degradation by modulating the MAPK pathway and its downstream effectors, MMP-1 and AP-1, thereby remodeling the ECM and exerting potent anti-aging effects. Importantly, the results suggested a potential self-amplifying feedback loop between ECM disruption and fibroblast senescence. SASP fibroblasts secreted inflammatory cytokines and proteases, including IL-6, MMP-1, and TGF-β, which accelerated ECM degradation. Loss of mechanical support and adhesion sites further promoted senescence and reinforced SASP. SF disrupted this pathological cycle by reconstructing the ECM and restoring the function of fibroblasts. Overall, these findings provide strong evidence for the therapeutic potential of SF in skin anti-aging interventions, establishing a dual-targeting strategy against fibroblast senescence and ECM imbalance.

## 4 Conclusions

SF is renowned for its exceptional biocompatibility and innate ability to promote collagen and elastin synthesis, as well as wound healing and tissue regeneration. Furthermore, its capacity to self-assemble into fibrous scaffolds provides critical structural support for cells, actively improving the skin's microenvironment. Despite these established advantages, its precise role in modulating fibroblast senescence and restoring ECM homeostasis remained elusive, limiting its clinical translation as a regenerative anti-aging agent. This study systematically explores the application of SF in skin anti-aging and its underlying molecular mechanisms, revealing that SF restores ECM homeostasis by modulating the function of fibroblasts. Specifically, SF provides a suitable microenvironment for the adhesion and proliferation of fibroblasts, reducing the accumulation of SASP factors and facilitating the transition of fibroblasts from a senescent to a functional state. SF improves the skin microenvironment by attenuating ROS and suppressing MMP expression via modulation of the ROS–MAPK–AP-1–MMP signaling axis, thereby delaying collagen degradation in aged skin. Compared to existing injectable materials such as PLLA microspheres, HA Pro, and Col III, SF can regulate ECM remodeling and exhibit more effective anti-aging outcomes while avoiding fibrosis and the adverse effects of inflammation-mediated collagen regeneration.

However, this study has some limitations that need to be addressed. First, this study mainly focuses on the short-term anti-aging effects of SF. Future research should extend the experimental period (1-2 years) to evaluate its long-term effects and potential risks. Second, while the study reveals SF regulates collagen degradation through the MAPK-AP-1-MMP pathway, further clarification of specific targets is required. Future studies could use gene editing technologies (e.g., CRISPR-Cas9) or proteomics to explore its intracellular mechanisms. Third, although SF has shown significant effects in laboratory and animal models, it still faces challenges in clinical applications, such as formulation stability, injection techniques, and individual patient differences. Further clinical trials are needed to verify its safety and efficacy in humans. Moreover, the UVB photoaging model used in this study does not fully replicate the complex processes of chronological aging, which involves cumulative cellular senescence and gradual ECM degradation. These aspects should be addressed in future research to reflect the natural aging process better.

In conclusion, this study systematically elucidates the anti-aging mechanisms of SF, highlighting its ability to restore ECM homeostasis and reactivate senescent fibroblasts via a non-inflammatory pathway. These findings offer new mechanistic insights and provide a solid foundation for the clinical translation of SF-based injectable biomaterials in aesthetic and regenerative medicine.

## CRediT authorship contribution statement

**Jialing Cheng:** Writing – original draft, Validation, Methodology, Investigation. **Guo Bao:** Writing – original draft, Methodology, Investigation, Funding acquisition. **Demin Lin:** Validation, Methodology. **Hongliang Wang:** Writing – review & editing. **Yanfang Yang:** Writing – review & editing. **Youbai Chen:** Writing – review & editing, Supervision, Conceptualization. **Meiying Ning:** Writing – review & editing, Supervision, Funding acquisition, Conceptualization. **Jun Ye:** Writing – review & editing, Supervision, Funding acquisition, Conceptualization. **Yuling Liu:** Writing – review & editing, Supervision, Conceptualization.

## Ethics approval and consent to participate

All animal experiments were approved by the Animal Care and Welfare Committee of the Institute of Materia Medical, Chinese Academy of Medical Sciences & Peking Union Medical College (Nos. 00001560, 00001823, 00001834, and 00002040).

## Declaration of Competing Interest

The authors declare no conflict of interest.

## References

[1] Sriram R., Gopal V. (2025). Mechanistic Insights on Skin ageing and Dermatologic Interventions to Slow Ageing Process. Indian Journal of Dermatology.

[2] Hajialiasgary Najafabadi A., Soheilifar M.H., Masoudi-Khoram N. (2024). Exosomes in skin photoaging: biological functions and therapeutic opportunity. Cell Commun Signal.

[3] Li F., Zhi J., Zhao R., Sun Y., Wen H., Cai H., Chen W., Jiang X., Bai R. (2024). Discovery of matrix metalloproteinase inhibitors as anti-skin photoaging agents. Eur. J. Med. Chem..

[4] S. Samizadeh, S. Samizadeh, Dermal Fillers: Understanding the Fundamentals, in: S. Samizadeh (Ed.), Non-Surgical Rejuvenation of Asian Faces, Springer International Publishing, Cham, 2022, pp. 253-265.

[5] Gao Q., Duan L., Feng X., Xu W. (2021). Superiority of poly(L-lactic acid) microspheres as dermal fillers. Chin. Chem. Lett..

[6] Oh H., Lee S., Na J., Kim J.H. (2021). Comparative Evaluation of Safety and Efficacy of a Novel Hyaluronic Acid-polynucleotide/Poly-L-lactic Acid Composite Dermal Filler. Aesthetic Plast Surg.

[7] Yu H., Zhang Y.-l., Chen Y., Dong Y.-x., Hong W.-j., Luo S.-k. (2025). In Vivo Effectiveness of Poly-L-Lactic Acid Microsphere Dermal Fillers in Stimulating Collagen Synthesis. Aesthetic Plastic Surgery.

[8] Wu S.-Y., Tsai C.-C., Tsai W.-B. (2025). From Bench to Clinic: Crosslinking Approaches for Next-Generation Collagen Fillers. Adv. Polym. Technol..

[9] He Q., Feng T., Xie Y., Swamiappan S., Zhou Y., Zhou Y., Zhou H., Peng X. (2025). Recent advances in the development and application of Cell-Loaded collagen scaffolds. Int. J. Mol. Sci..

[10] Puranik N., Tiwari S., Kumari M., Yadav S.K., Dhakal T., Song M. (2025). Advanced Bioactive Polymers and Materials for Nerve Repair: Strategies and Mechanistic Insights. Journal of Functional Biomaterials.

[11] Salehi M., Arabpour Z., Zamani S., Alizadeh M., Gharibshahiyan M., Rezvani M., Aldaghi N., Yekesadat S.M., Djalilian A.R. (2025). Corneal bioengineering via electrospun nanofibers and nanoparticles. J. Biomater. Appl..

[12] Lee J., Park S., Lee S., Kweon H.Y., Jo Y.Y., Kim J., Chung J.H., Seonwoo H. (2023). Development of Silk Fibroin-Based Non-Crosslinking Thermosensitive Bioinks for 3D Bioprinting. Polymers (Basel).

[13] Chen Y., Lyu R., Wang J., Cheng Q., Yu Y., Yang S., Mao C., Yang M. (2023). Metal-Organic Frameworks Nucleated by Silk Fibroin and Modified with Tumor-Targeting Peptides for Targeted Multimodal Cancer Therapy. Adv Sci (Weinh).

[14] Liu J., Xie X., Wang T., Chen H., Fu Y., Cheng X., Wu J., Li G., Liu C., Liimatainen H., Zheng Z., Wang X., Kaplan D.L. (2023). Promotion of Wound Healing Using Nanoporous Silk Fibroin Sponges. ACS Appl. Mater. Interfaces.

[15] Zheng H., Zuo B. (2021). Functional silk fibroin hydrogels: preparation, properties and applications. J. Mater. Chem. B.

[16] Zhu J., Du Y., Backman L.J., Chen J., Ouyang H., Zhang W. (2025). Cellular Interactions and Biological Effects of Silk Fibroin: Implications for Tissue Engineering and Regenerative Medicine. Small.

[17] Cheng J., Chen Z., Lin D., Yang Y., Bai Y., Wang L., Li J., Wang Y., Wang H., Chen Y., Ye J., Liu Y. (2025). A high clinically translatable strategy to anti-aging using hyaluronic acid and silk fibroin co-crosslinked hydrogels as dermal regenerative fillers. Acta Pharm. Sin. B.

[18] Aldahish A., Shanmugasundaram N., Vasudevan R., Alqahtani T., Alqahtani S., Mohammad Asiri A., Devanandan P., Thamaraikani T., Vellapandian C., Jayasankar N. (2024). Silk Fibroin Nanofibers: Advancements in Bioactive Dressings through Electrospinning Technology for Diabetic Wound Healing. Pharmaceuticals.

[19] Radulescu D.-M., Andronescu E., Vasile O.R., Ficai A., Vasile B.S. (2024). Silk fibroin-based scaffolds for wound healing applications with metal oxide nanoparticles. J. Drug Delivery Sci. Technol..

[20] Hua J., Huang R., Yu M., You R., Wang L., Yan S., Huang Y., Zhang Q. (2025). High-performance silk fibroin/hyaluronic acid interpenetrating network hydrogel microneedles for diabetes management. Int. J. Biol. Macromol..

[21] Oh H., Lee S., Na J., Kim J.H. (2021). Comparative Evaluation of Safety and Efficacy of a Novel Hyaluronic Acid-polynucleotide/Poly-L-lactic Acid Composite Dermal Filler. Aesthetic Plastic Surgery.

[22] Sedush N.G., Kalinin K.T., Azarkevich P.N., Gorskaya A.A., Hano C. (2023). Physicochemical Characteristics and Hydrolytic Degradation of Polylactic Acid Dermal Fillers: A Comparative Study. Cosmetics.

[23] Ray S., Adelnia H., Ta H.T. (2021). Collagen and the effect of poly-l-lactic acid based materials on its synthesis. Biomater. Sci..

[24] D.M. Reilly, J. Lozano, Skin collagen through the lifestages: importance for skin health and beauty, Plastic and Aesthetic Research 8 (2021).

[25] Ballin A.C., Brandt F.S., Cazzaniga A. (2015). Dermal fillers: an update. Am J Clin Dermatol.

[26] Guo J., Fang W., Wang F. (2023). Injectable fillers: current status, physicochemical properties, function mechanism, and perspectives. RSC Adv.

[27] Bachelor M.A., Bowden G.T. (2004). UVA-mediated activation of signaling pathways involved in skin tumor promotion and progression. Semin. Cancer Biol..

[28] Zorina A., Zorin V., Isaev A., Kudlay D., Vasileva M., Kopnin P. (2023). Dermal Fibroblasts as the Main Target for Skin Anti-Age Correction Using a Combination of Regenerative Medicine Methods. Curr Issues Mol Biol.

[29] Suryadevara V., Hudgins A.D., Rajesh A., Pappalardo A., Karpova A., Dey A.K., Hertzel A., Agudelo A., Rocha A., Soygur B., Schilling B., Carver C.M., Aguayo-Mazzucato C., Baker D.J., Bernlohr D.A., Jurk D., Mangarova D.B., Quardokus E.M., Enninga E.A.L., Schmidt E.L., Chen F., Duncan F.E., Cambuli F., Kaur G., Kuchel G.A., Lee G., Daldrup-Link H.E., Martini H., Phatnani H., Al-Naggar I.M., Rahman I., Nie J., Passos J.F., Silverstein J.C., Campisi J., Wang J., Iwasaki K., Barbosa K., Metis K., Nernekli K., Niedernhofer L.J., Ding L., Wang L., Adams L.C., Ruiyang L., Doolittle M.L., Teneche M.G., Schafer M.J., Xu M., Hajipour M., Boroumand M., Basisty N., Sloan N., Slavov N., Kuksenko O., Robson P., Gomez P.T., Vasilikos P., Adams P.D., Carapeto P., Zhu Q., Ramasamy R., Perez-Lorenzo R., Fan R., Dong R., Montgomery R.R., Shaikh S., Vickovic S., Yin S., Kang S., Suvakov S., Khosla S., Garovic V.D., Menon V., Xu Y., Song Y., Suh Y., Dou Z., Neretti N. (2024). SenNet recommendations for detecting senescent cells in different tissues. Nat. Rev. Mol. Cell Biol..

[30] Fisher G.J., Wang B., Cui Y., Shi M., Zhao Y., Quan T., Voorhees J.J. (2023). Skin aging from the perspective of dermal fibroblasts: the interplay between the adaptation to the extracellular matrix microenvironment and cell autonomous processes. J Cell Commun Signal.

[31] Alqahtani S., Alqahtani T., Venkatesan K., Sivadasan D., Ahmed R., Sirag N., Elfadil H., Abdullah Mohamed H., T A.H., Elsayed Ahmed R., Muralidharan P., Paulsamy P. (2025). SASP Modulation for Cellular Rejuvenation and Tissue Homeostasis: Therapeutic Strategies and Molecular Insights. Cells.

[32] Lee Y.I., Lee S.G., Jung I., Suk J., Lee M.H., Kim D.U., Lee J.H. (2022). Effect of a Topical Collagen Tripeptide on Antiaging and Inhibition of Glycation of the Skin: A Pilot Study. Int. J. Mol. Sci..

[33] Cavinato M., Jansen-Durr P. (2017). Molecular mechanisms of UVB-induced senescence of dermal fibroblasts and its relevance for photoaging of the human skin. Exp Gerontol.

[34] Nikolov A., Popovski N., Hristova I. (2020). Collagenases MMP-1, MMP-13, and Tissue Inhibitors TIMP-1, TIMP-2: Their Role in Healthy and Complicated Pregnancy and Potential as Preeclampsia Biomarkers—A Brief Review. Applied Sciences.

