# Supplementary material for: Silk Fibroin Counteracts Fibroblast Senescence to Restore ECM Homeostasis in Aged Skin
Source: Bioact Mater. 2026 Jan 6;58:666–84. doi: 10.1016/j.bioactmat.2025.12.006 (PMC12808896; doi:10.1016/j.bioactmat.2025.12.006)
Supplement: Supplementary file 1 [file mmc1.docx]

**Supplemental figures of “Silk Fibroin Rejuvenates Fibroblasts to Drive ECM Remodeling in Skin Aging”**


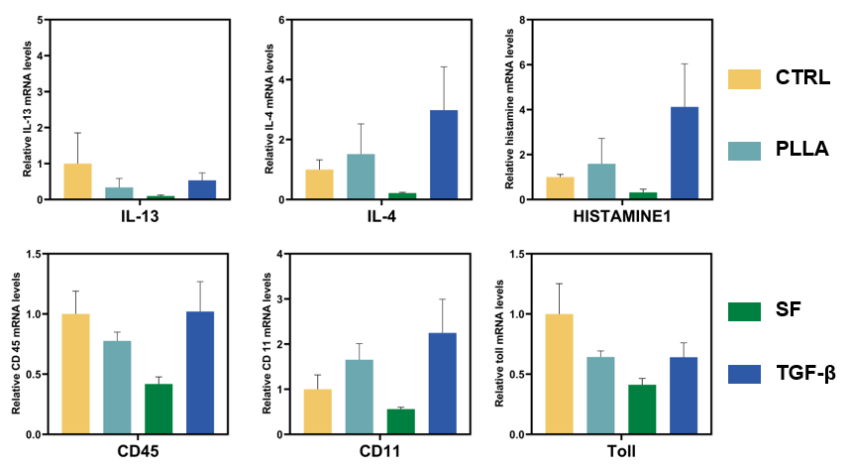


**Fig. S1** Inflammatory factor after co-culture with RAW+L929 cells of SF and PLLA.


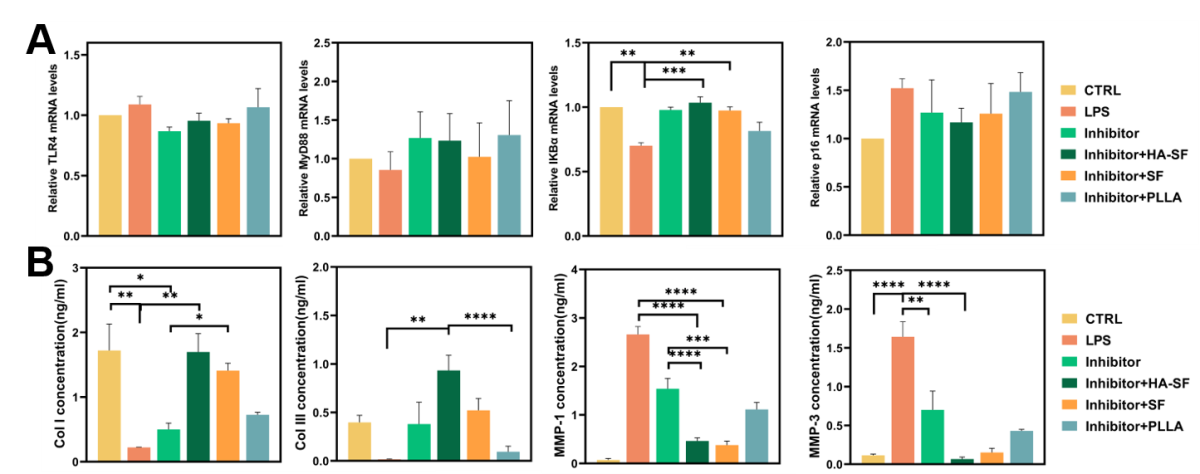


**Fig. S2** Result of NF-κB inhibition assay. (A) qPCR analysis of TLR4, MyD88, IκBα, and p16 in the HA-SF, SF, and PLLA groups following treatment with the NF-κB inhibitor. (B) ELISA measurements of Col I, Col III, MMP-1, and MMP-3.


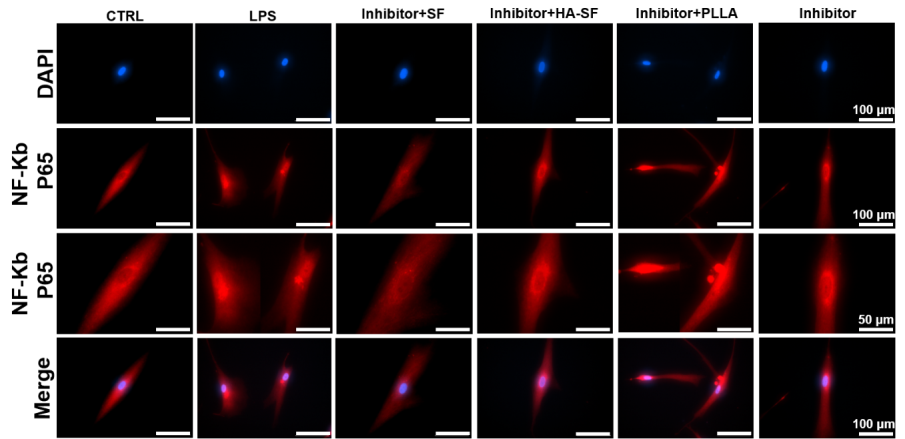

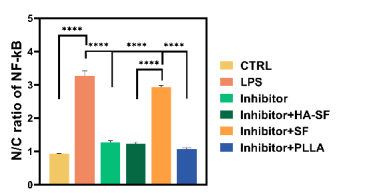


**Fig. S3** Effects of NF-κB inhibition on p-p65 nuclear translocation. Immunofluorescence staining and quantitative analysis of p65 nuclear translocation after addition of the NF-κB inhibitor.


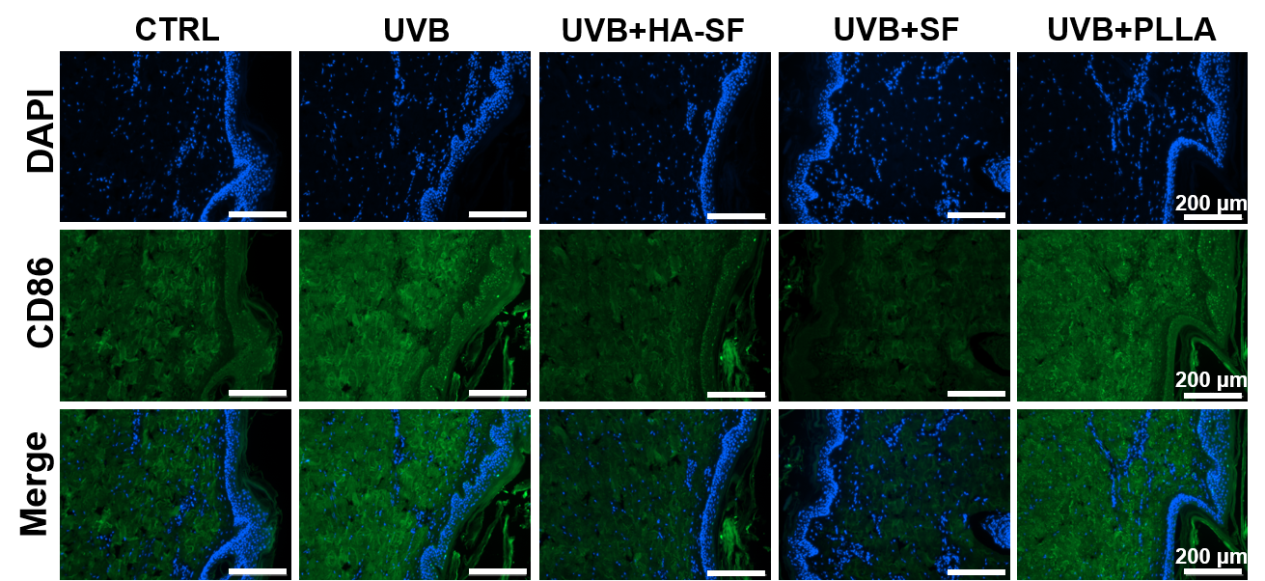


**Fig. S4** IF staining of CD86 in Bama minipig skin.


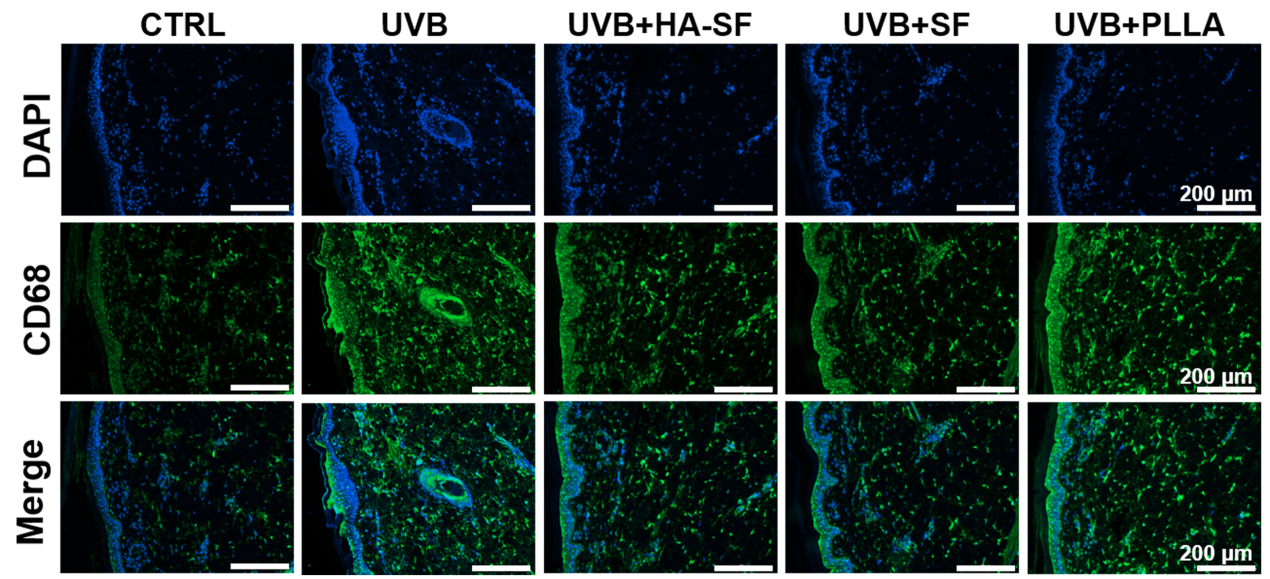


**Fig. S5** IF staining of CD68 in Bama minipig skin.


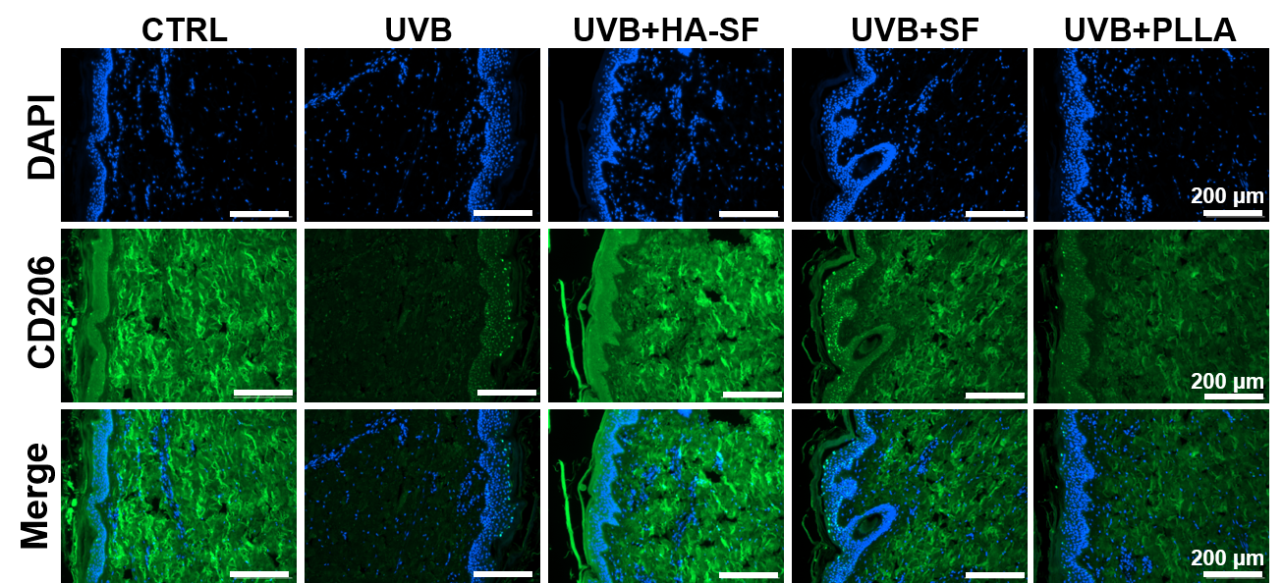


**Fig. S6** IF staining of CD206 in Bama minipig skin.


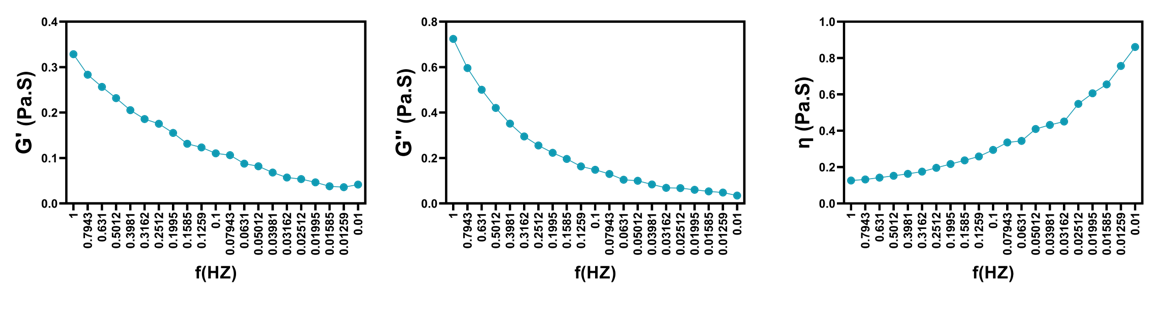


**Fig. S7** Frequency- and strain-dependent rheology of the HA-SF composite. G′, G″, and viscosity were measured across varying frequencies and strains, reflecting the material’s viscoelastic stability and injectability.


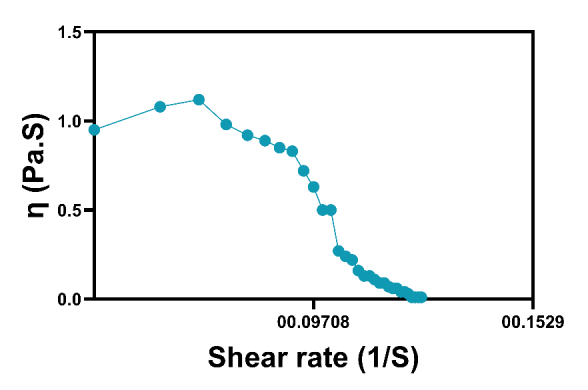


**Fig. S8** Shear rate–dependent viscosity of the HA-SF composite. Viscosity was measured over a range of shear rates to evaluate the shear-thinning behavior.


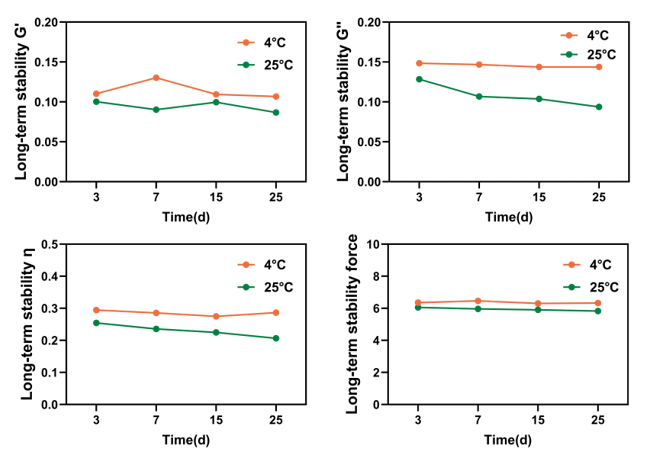


**Fig. S9** Long-term stability of the HA-SF composite. Storage modulus (G′), loss modulus (G″), viscosity, and extrusion force were monitored at 4°C and 25°C to assess rheological and mechanical stability over time.


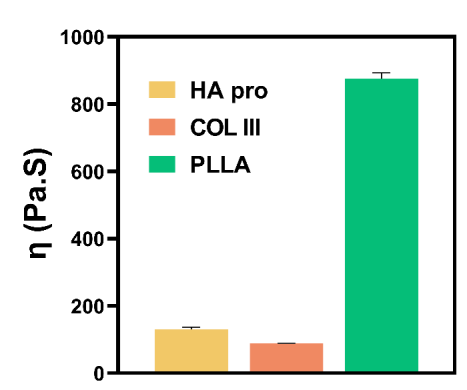

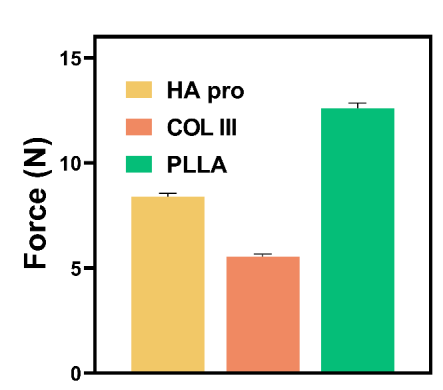


**Fig. S10 Comparison of viscosity and extrusion force among commercial products.**


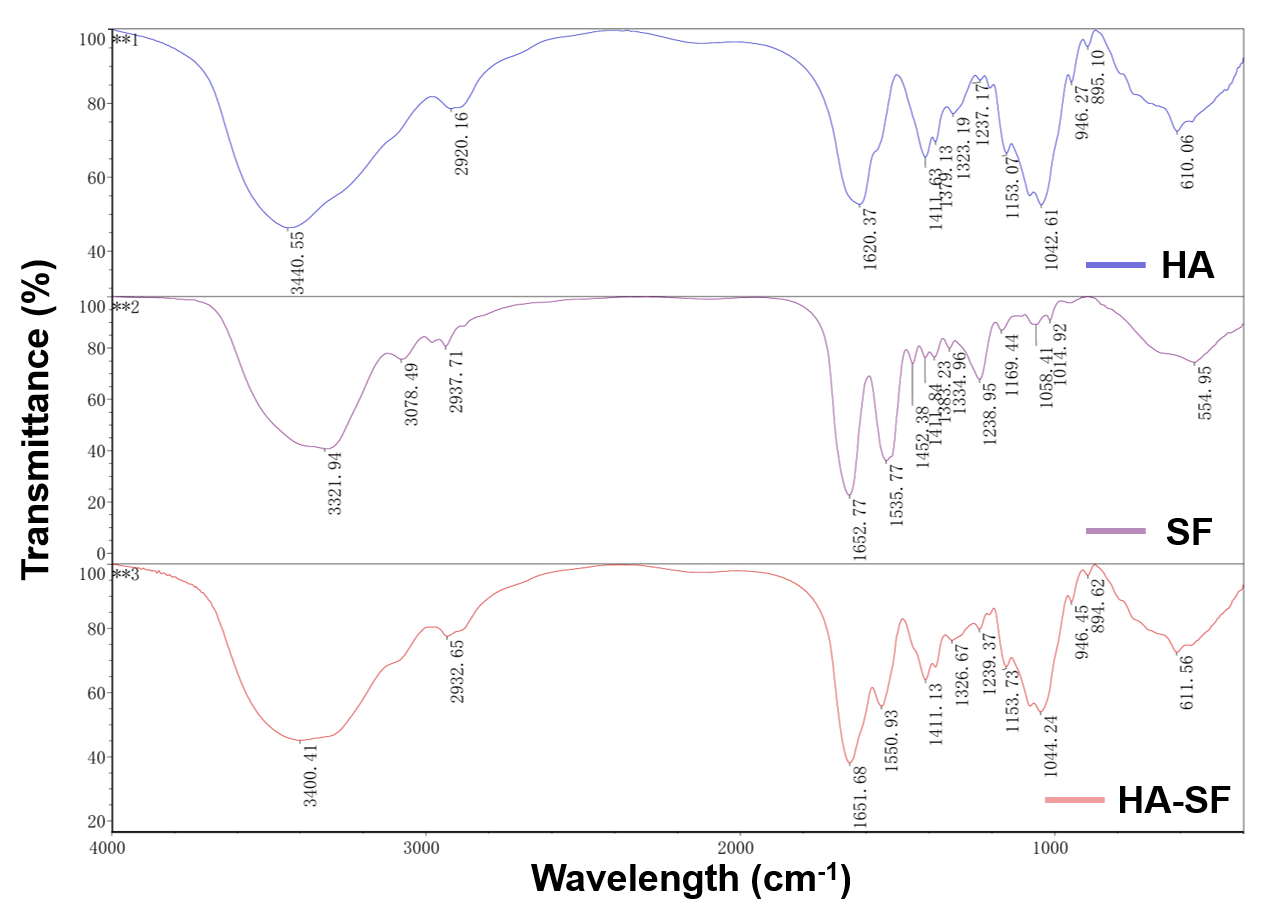

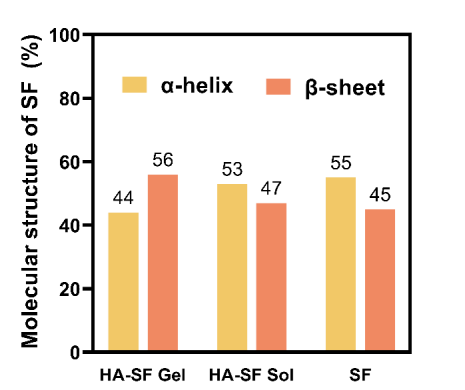


**Fig. S11** FTIR spectra of HA, SF, and the HA-SF composite, and quantitative analysis of the β-sheet structure.


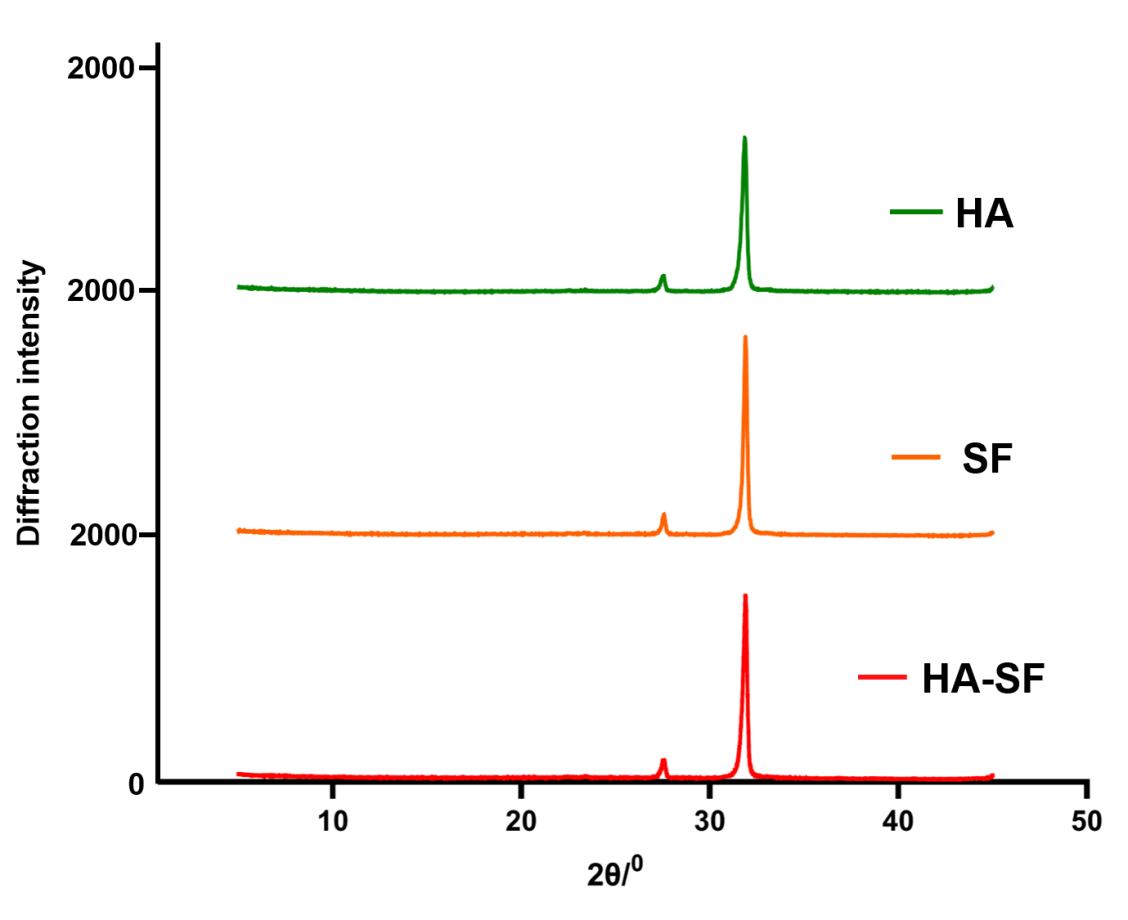


**Fig. S12** XRD analysis of HA, SF, and the HA-SF composite.
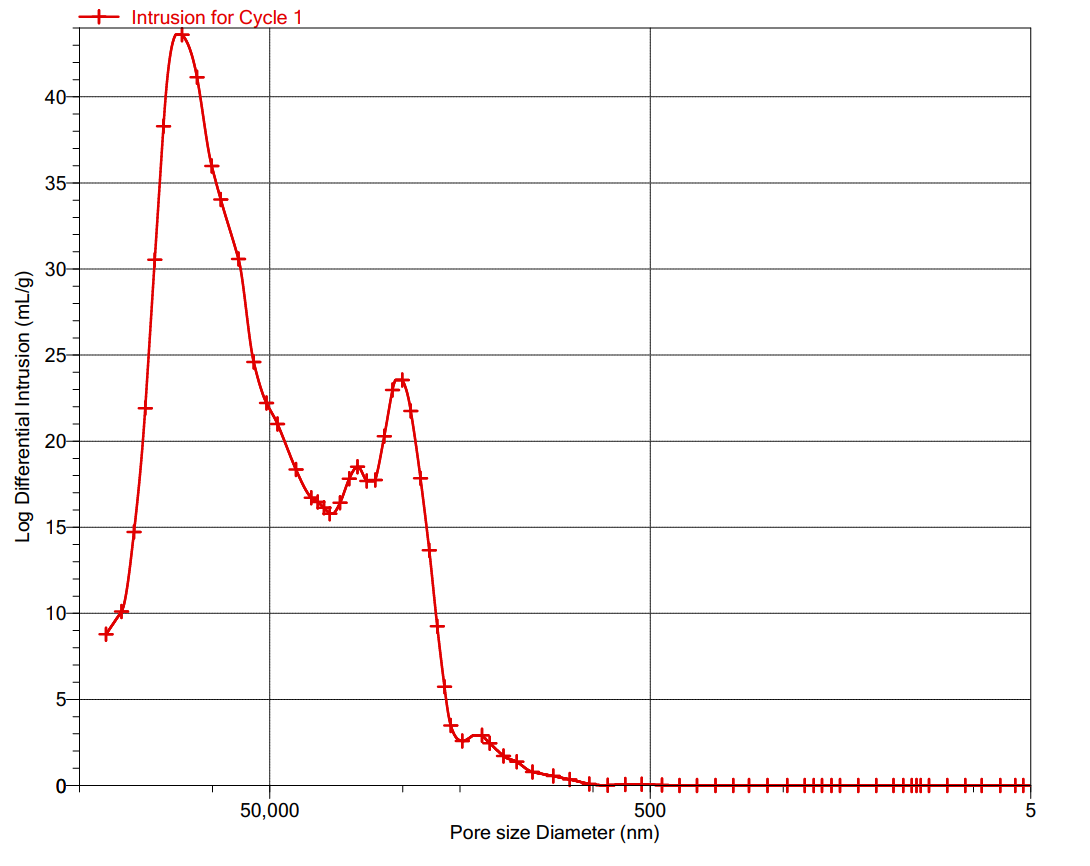


**Fig. S13** Log differential intrusion versus pore size of lyophilized SF.


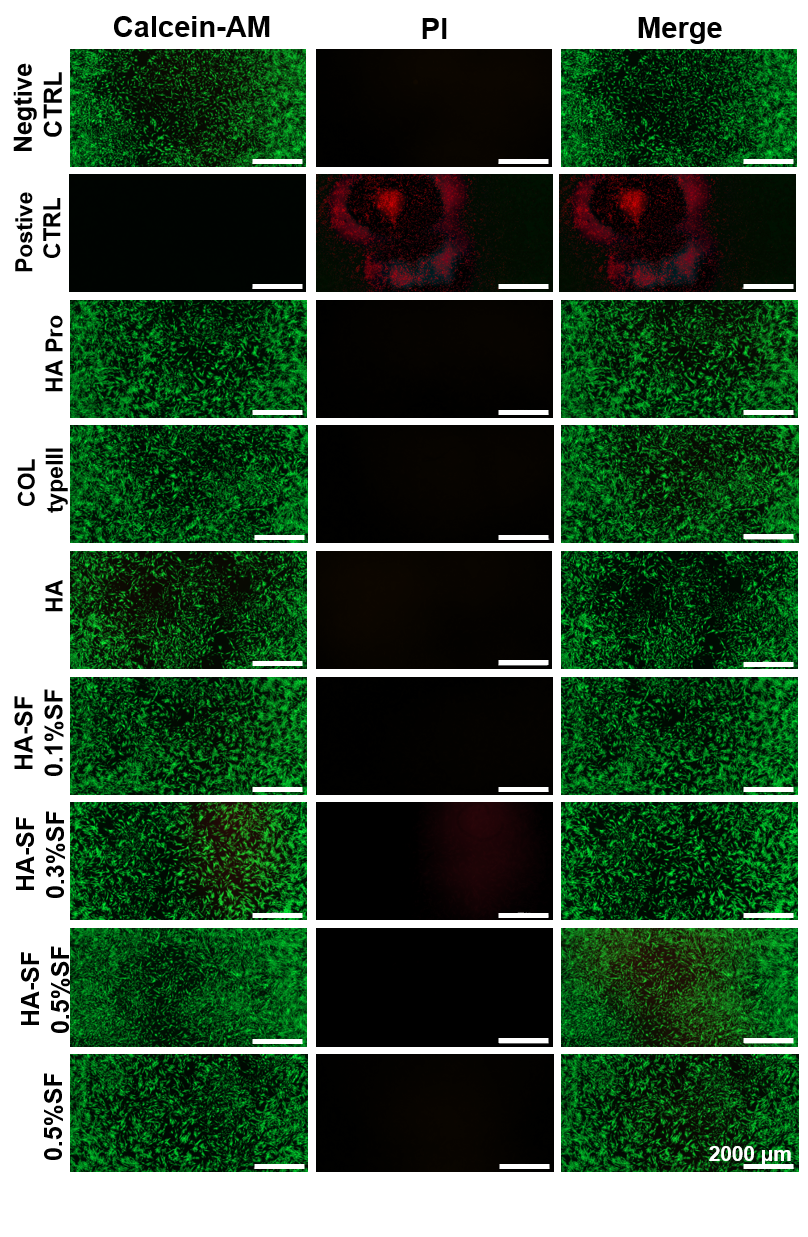


**Fig. S14** Live/dead staining after co-culture with ESF-HF-1 cells of HA Pro, Col III, HA, SF, HA-SF (0.1% SF), HA-SF (0.3% SF), HA-SF (0.5% SF).


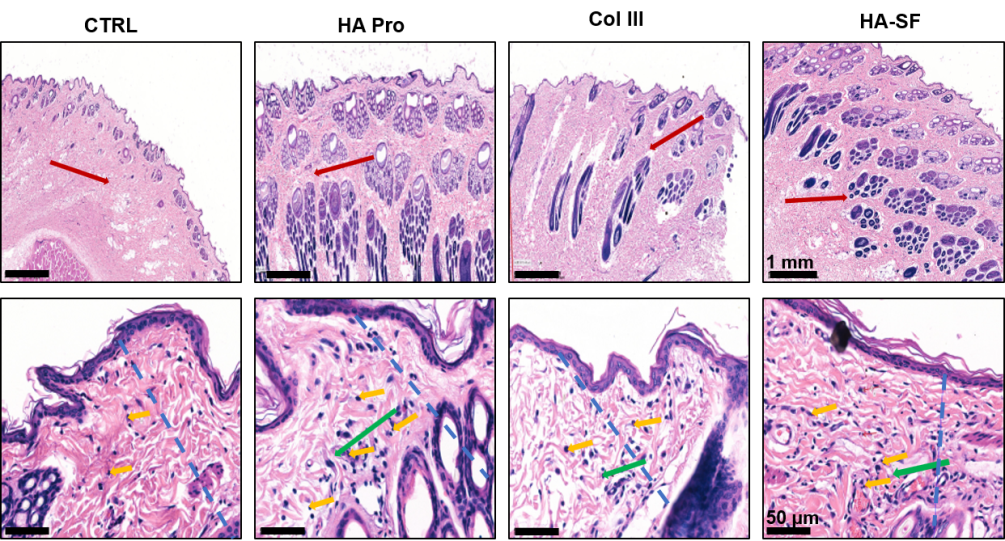


**Fig. S15** HE staining of the skin of New Zealand rabbits with HA-SF, HA Pro, and Col III injected for 4 w. Red arrows: skin dermis. Blue dotted line: dermal location. Green arrows: macrophages. Yellow cells: neutrophils.


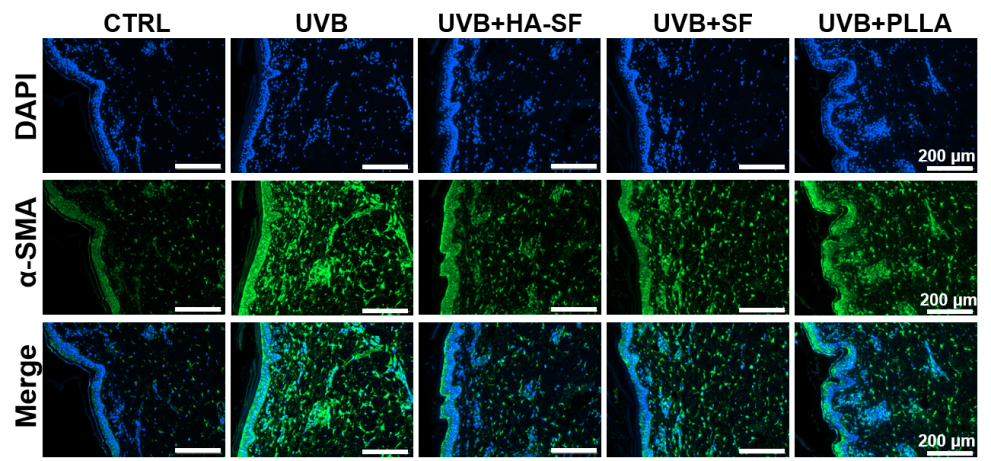


**Fig. S16** IF staining of α-SMA in Bama minipig skin.


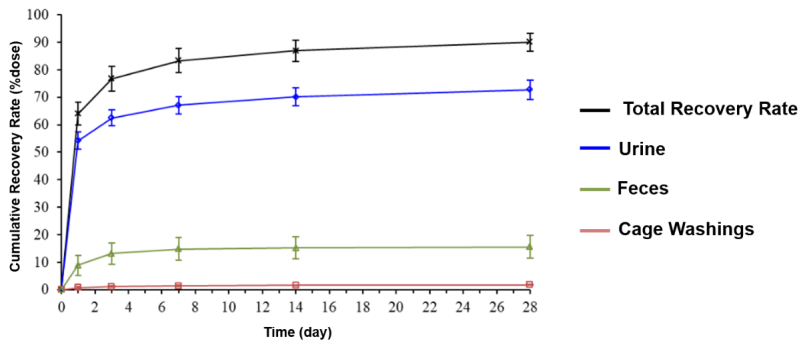


**Fig. S17** Radioactivity recovery profile of isotopically labeled HA-SF after implantation.


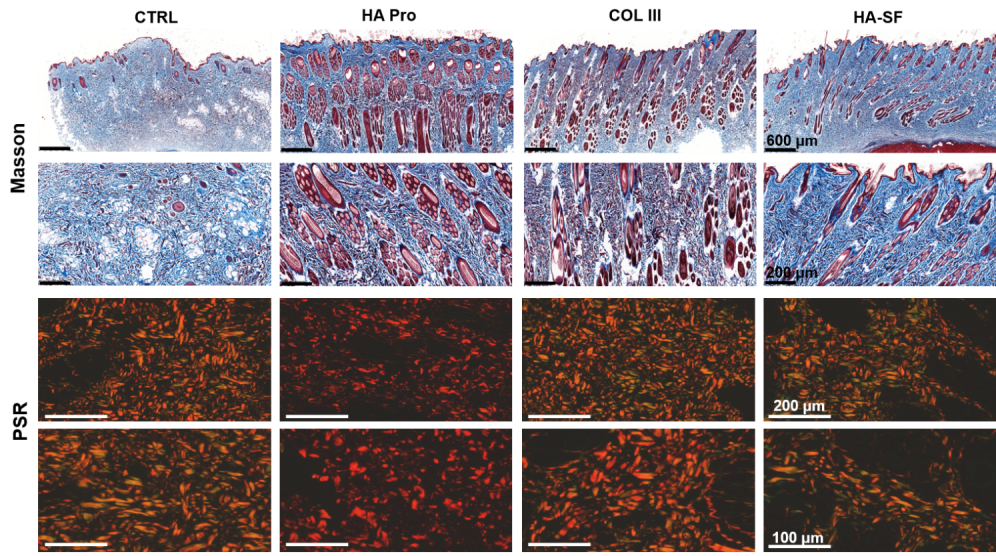


**Fig. S18** Masson staining and PSR staining of the skin of New Zealand rabbits with HA-SF, HA Pro, and Col III injected for 4 w.


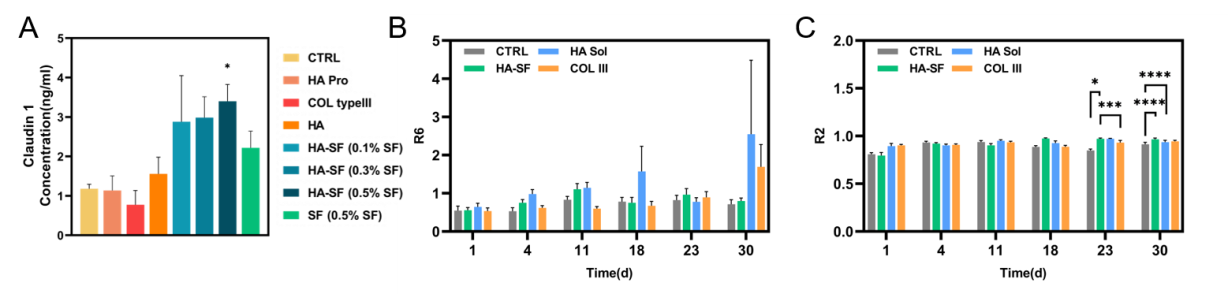


**Fig. S19** Efficacy study of HA-SF microneedles. (A) CLDN1 expression after co-culture of HA-SF with Hacat cells. (B) and (C) Elasticity curve index of HA-SF injected into the skin of New Zealand rabbits.


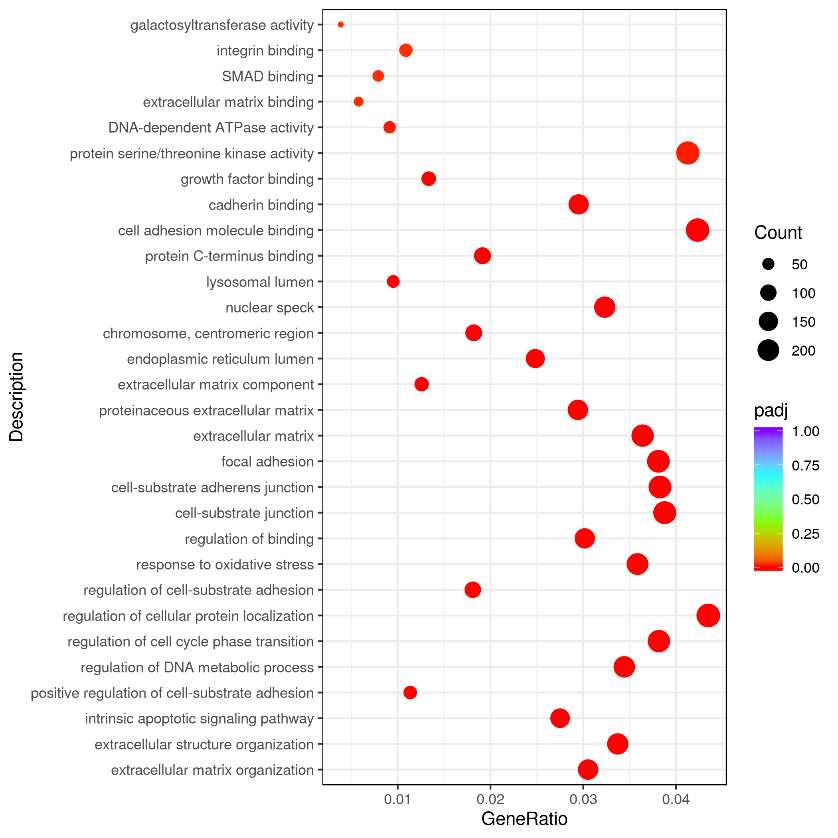

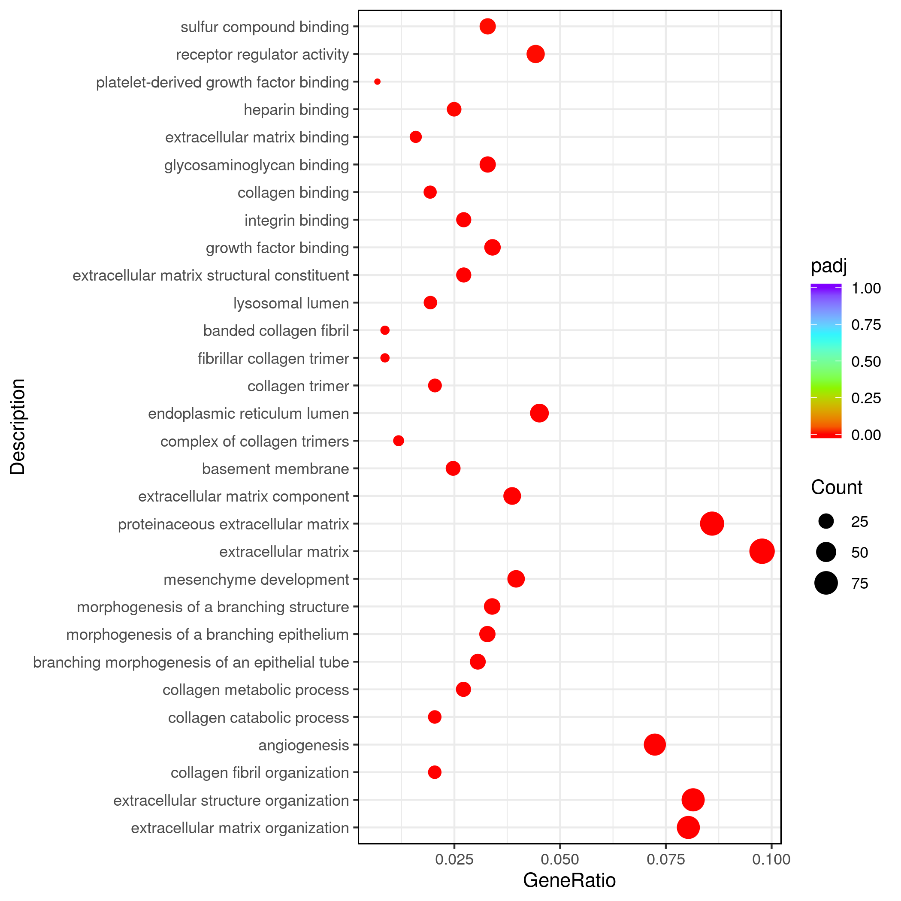


**Fig. S20** Functional Gene Ontology (GO) classification of DEPs in CTRL vs SF and UVB vs CTRL. The x-axis represents pathway annotation entries, and the y-axis shows the number of DEPs enriched in each pathway.


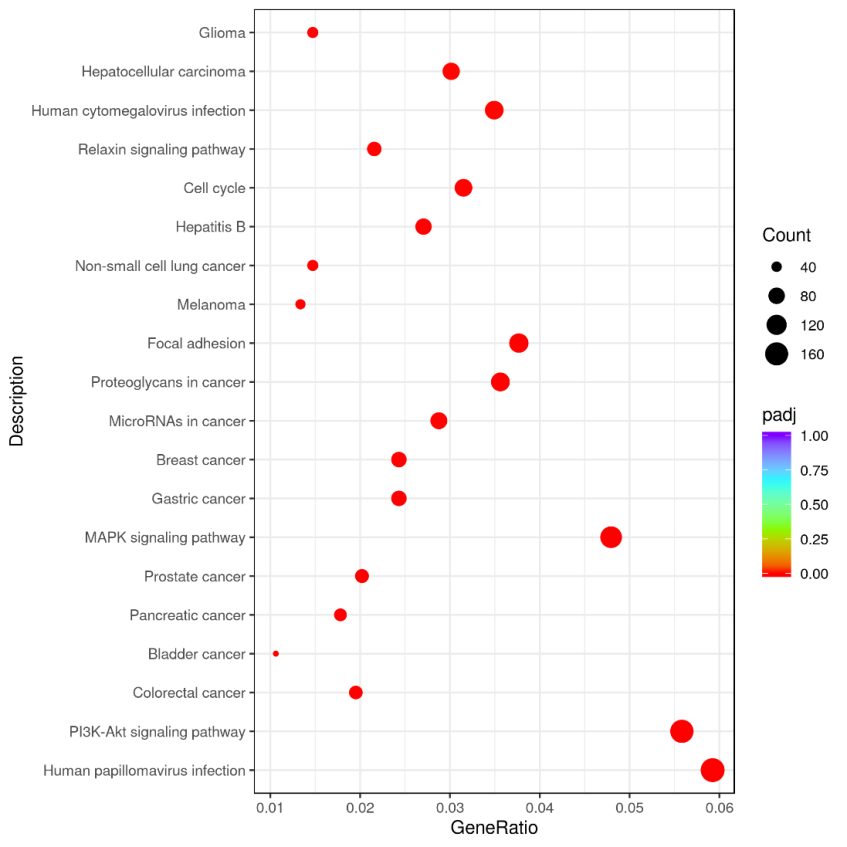

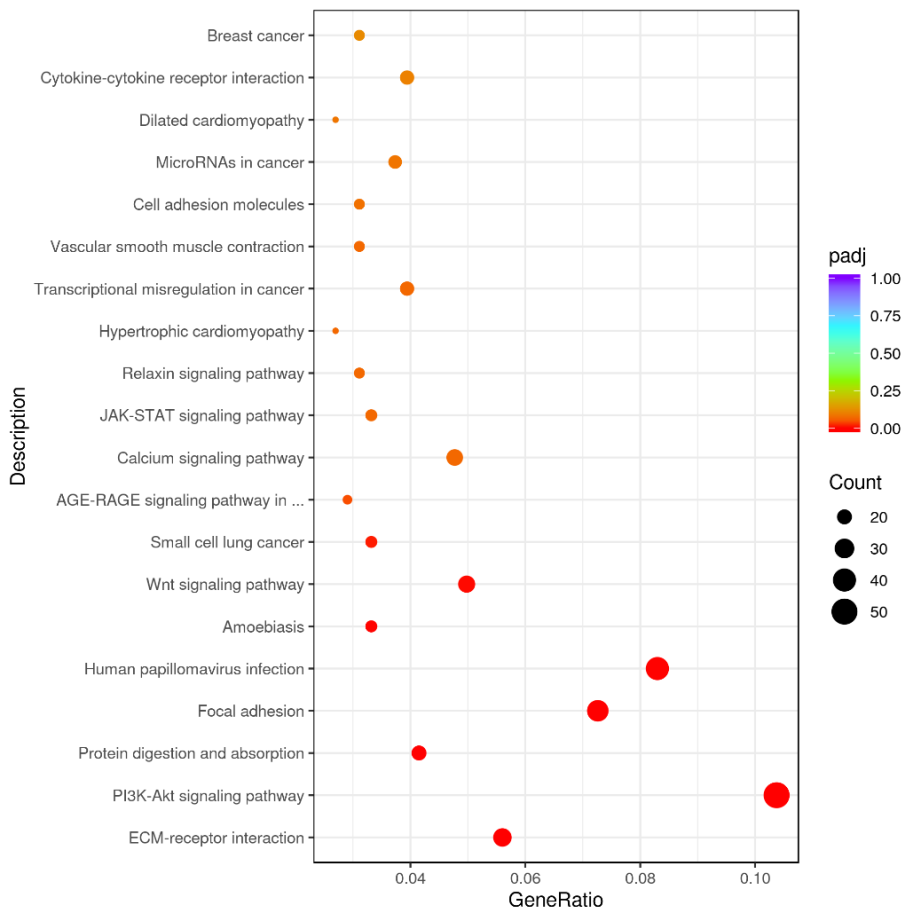


**Fig. S21** Kyoto Encyclopedia of Genes and Genomes (KEGG) pathway classification of DEPs in CTRL vs SF and UVB vs CTRL. The x-axis indicates the enrichment factor (Ratio), i.e., the number of DEPs annotated to each pathway divided by all identified proteins annotated to the same pathway. The larger the value, the greater the proportion of DEPs annotated to each pathway. Dot sizes represent the number of DEPs annotated to each pathway.


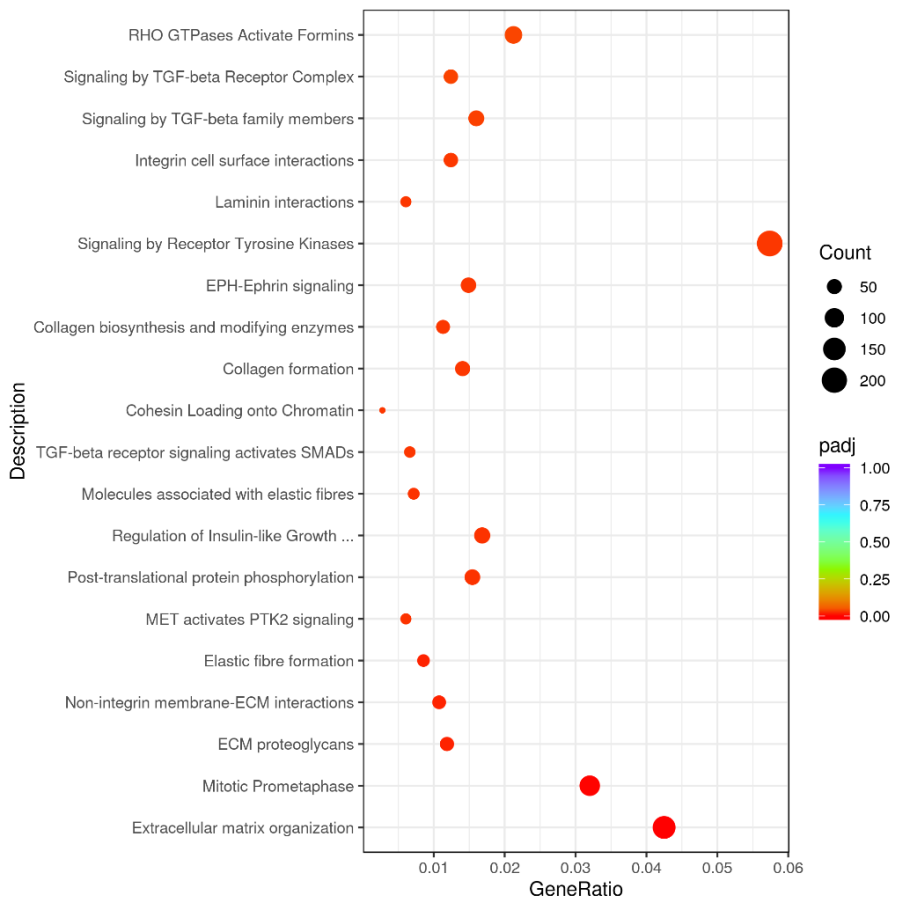

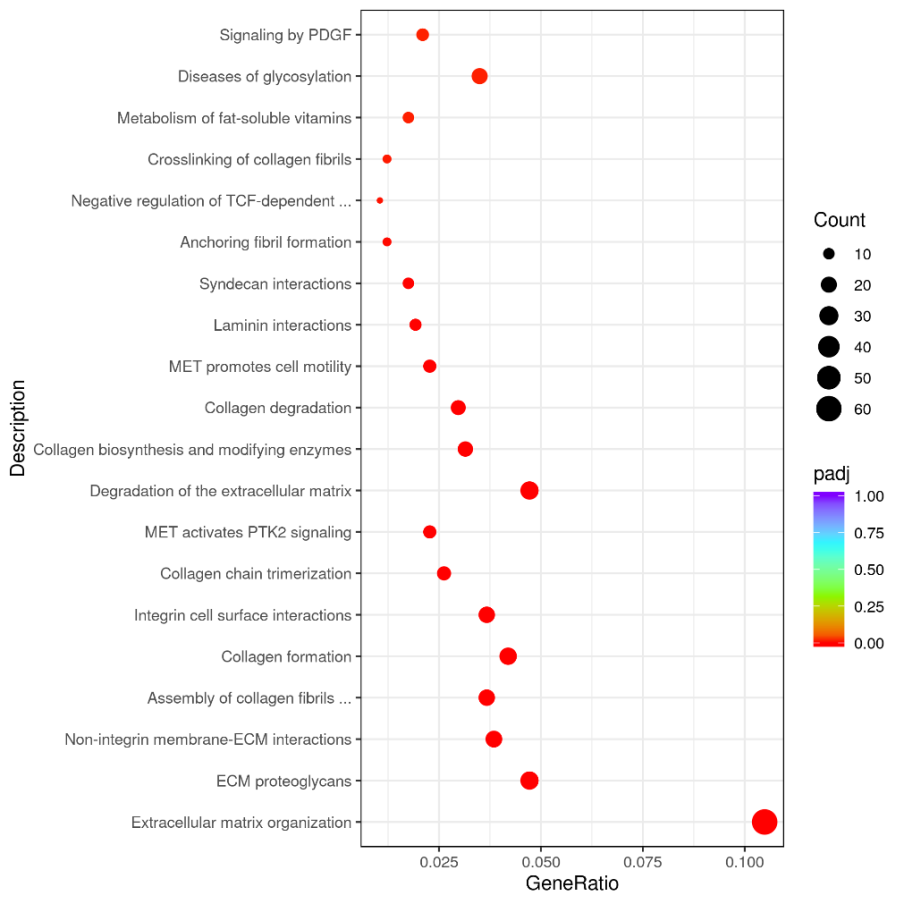


**Fig. S22** Reactome pathway classification of DEPs in CTRL vs SF and UVB vs CTRL. The x-axis indicates the enrichment factor (Ratio), i.e., the number of DEPs annotated to each pathway divided by all identified proteins annotated to the same pathway. The larger the value, the greater the proportion of DEPs annotated to each pathway. Dot sizes represent the number of DEPs annotated to each pathway.


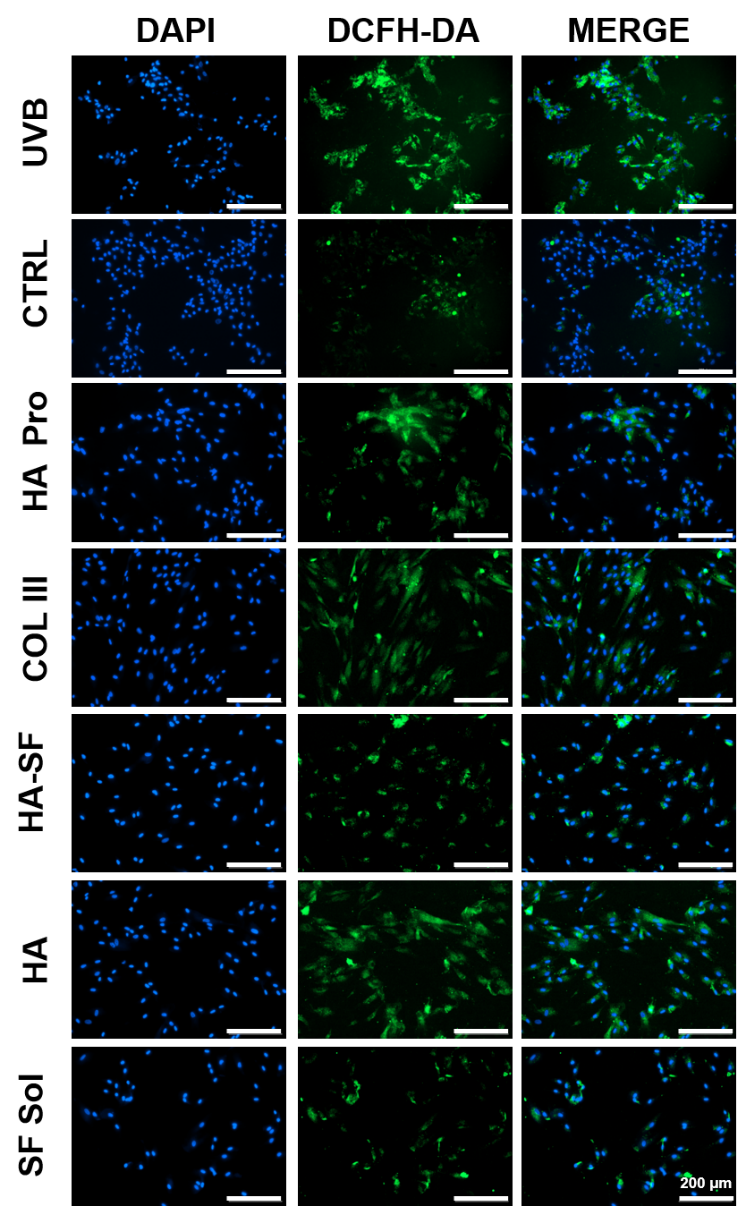


**Fig. S23** ROS staining of ESF-HF-1 cells after co-culture with HA Pro, Col III, HA, SF, HA-SF (0.1% SF), HA-SF (0.3% SF), and HA-SF (0.5% SF).


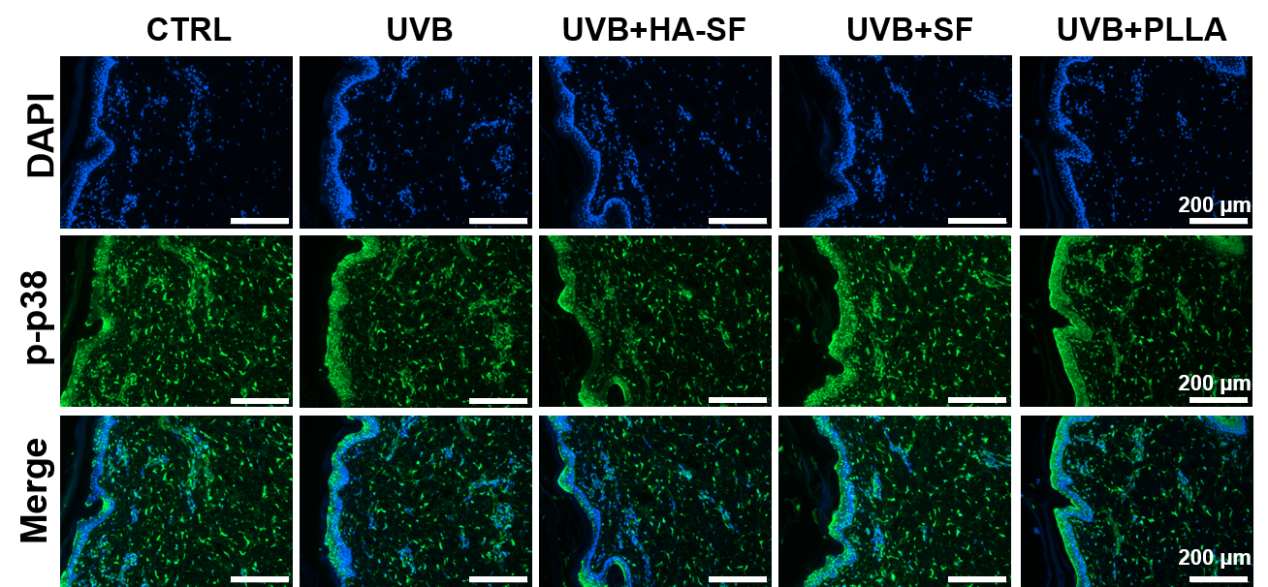


**Fig. S24** IF staining of p-p38 in Bama minipig skin.


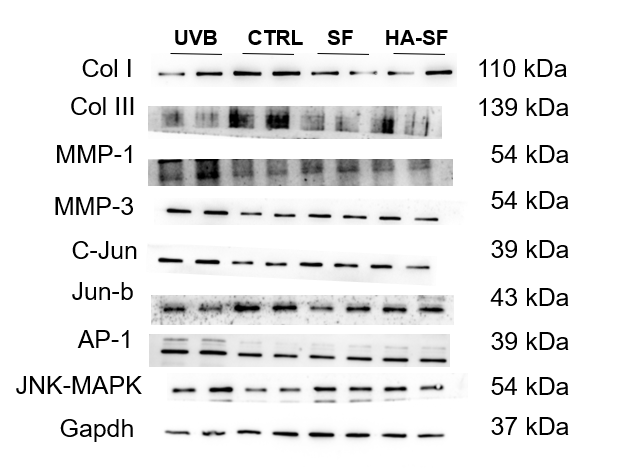


**Fig. S25** WB validation of SF regulation of the MAPK-AP-1-MMP pathway (n=4).


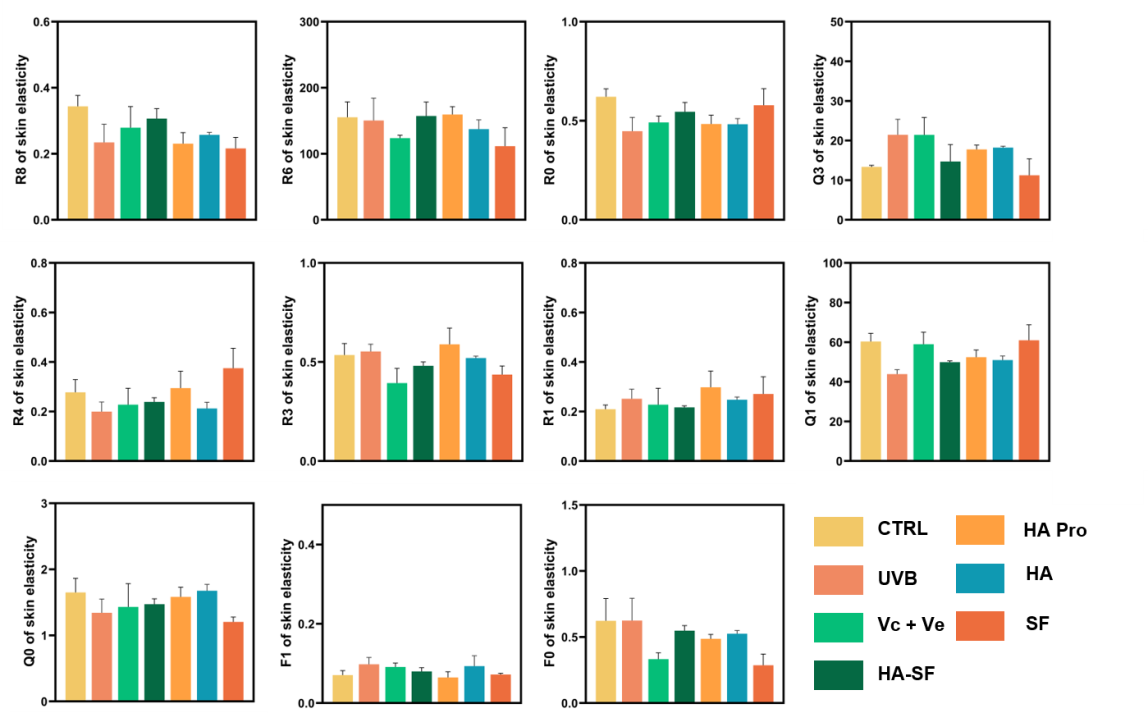


**Fig. S26** Elasticity curves of 3D skin-organoid after culturing for HA Pro, HA, SF, and HA-SF (0.5% SF).


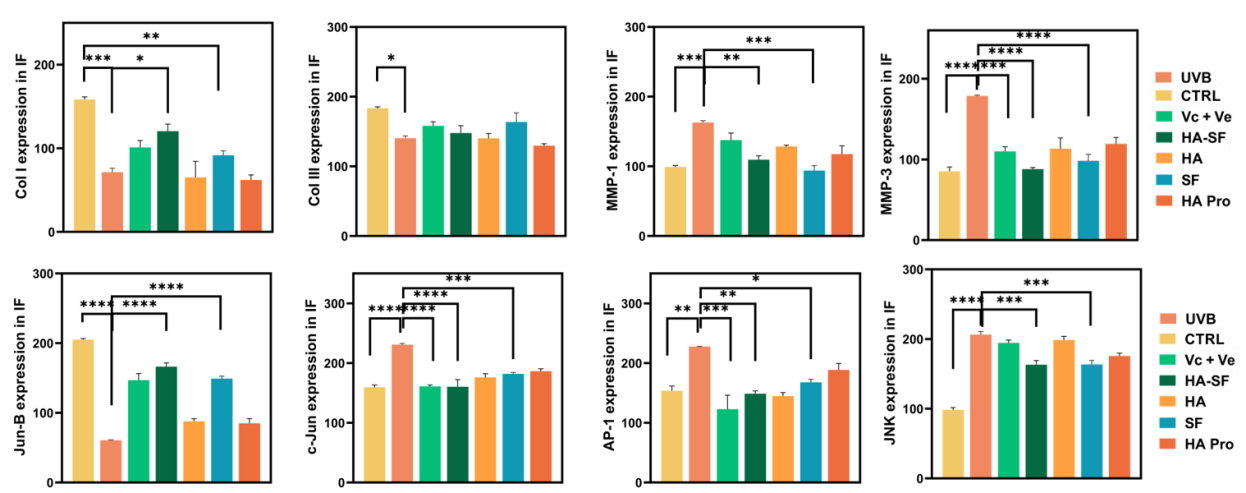


**Fig. S27** IF staining of 3D skin-organoid after culturing for HA Pro, HA, SF, and HA-SF (0.5% SF).


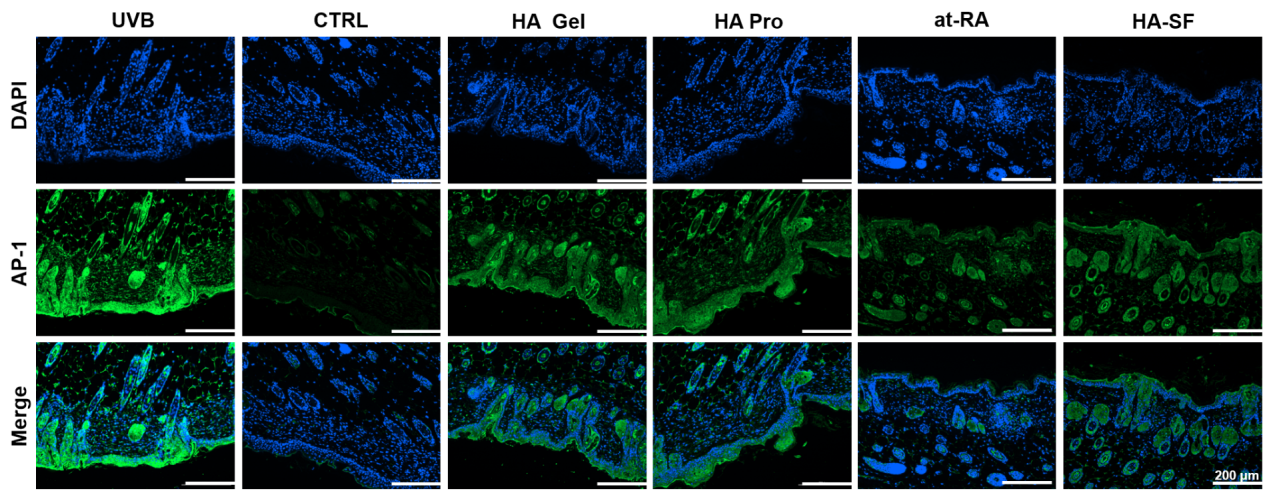


**Fig. S28** AP-1 expression in different groups (HA Pro, HA Gel, and HA-SF Gel) of UVB-induced nude mouse skin.


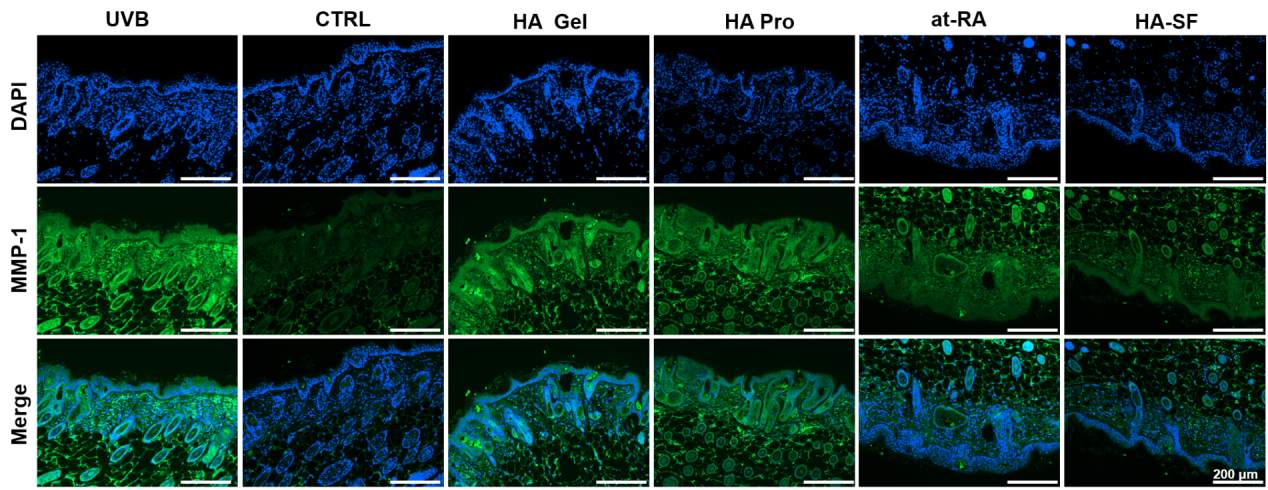


**Fig. S29**MMP-1 expression in different groups (HA Pro, HA Gel, and HA-SF Gel) of UVB-induced nude mouse skin.


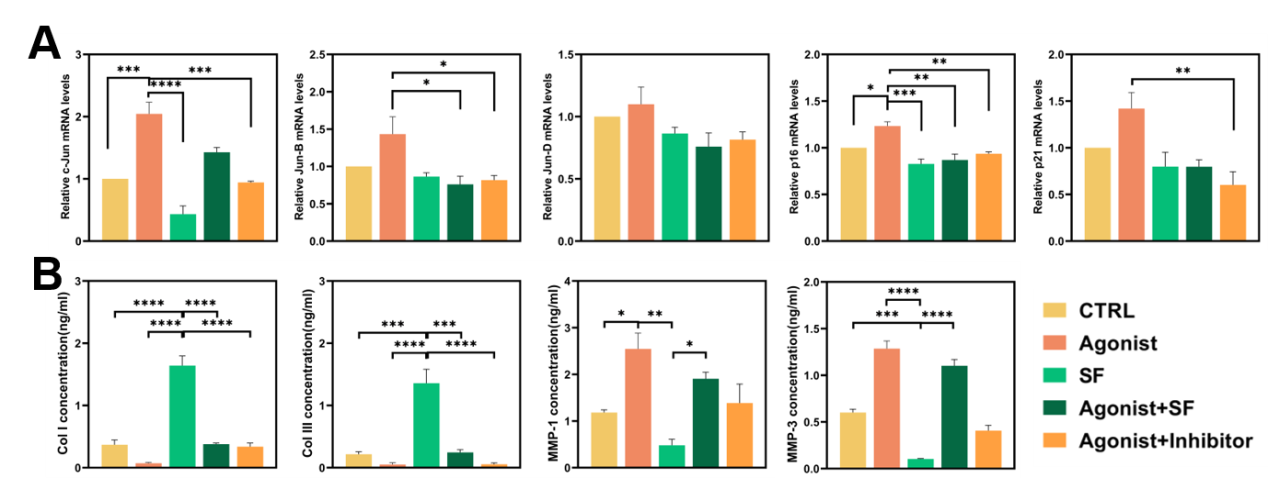


**Fig. S30 Result of JNK agonist assay.** (A) qPCR analysis of c-Jun, Jun-B, Jun-D, p16 and p21 in the SF, JNK agonist and Inhibitor groups following. (B) ELISA measurements of COl I, Col III, MMP-1, and MMP-3 in the SF, JNK agonist and Inhibitor groups.


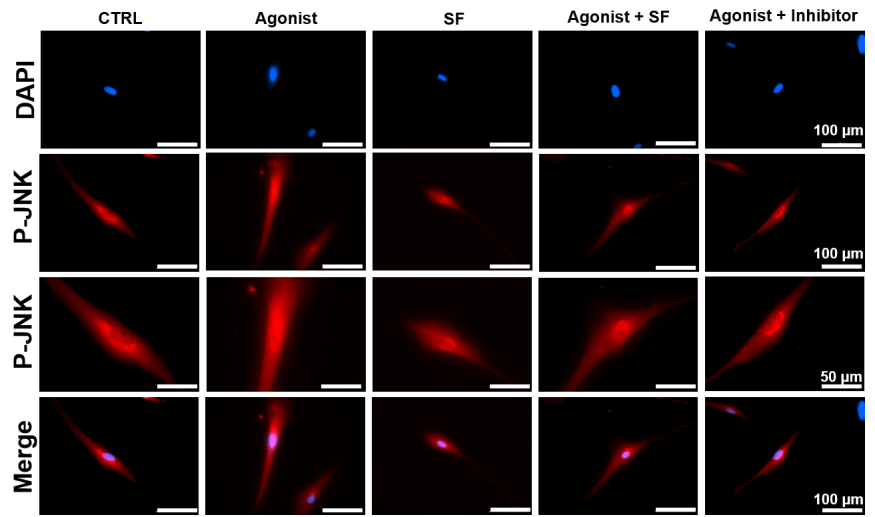

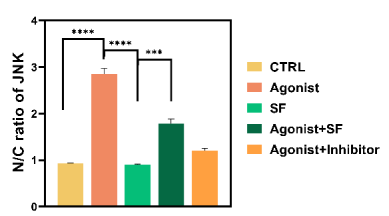


**Fig. S31 Effects of** **JNK Agonist on** **p-JNK nuclear translocation.** Immunofluorescence staining and quantitative analysis of p-JNK nuclear translocation after addition of the JNK Agonist.


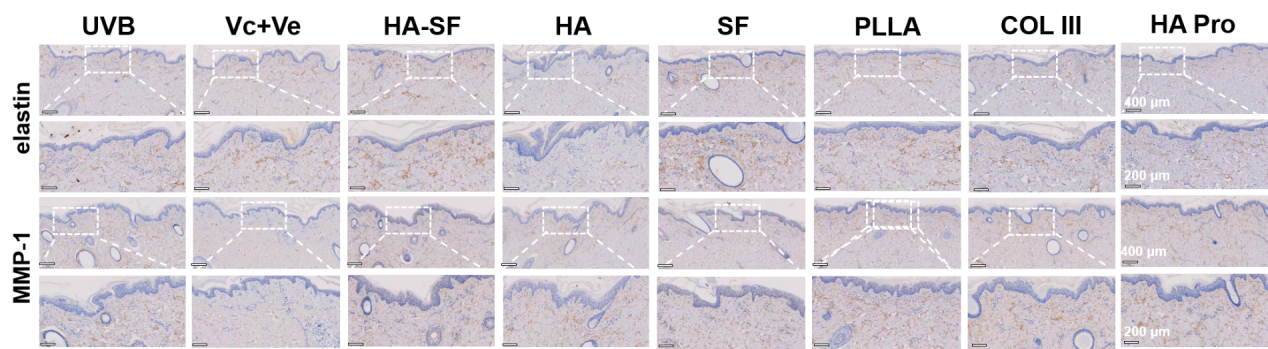


**Fig. S32** Elastin and MMP-1 expression of UVB-induced Bama pig model skin.

**Tab. S1** Q-PCR primer sequences throughout the study.

| Primer | Sequence（5’-3’） |  |
| --- | --- | --- |
| M-Collagen I-F | GGTGAGCCTGGTCAAACGG |  |
| M-Collagen I-R | ACTGTGTCCTTTCACGCCTTT |  |
| M-Collagen III-F | CTGTAACATGGAAACTGGGGAAA |  |
| M-Collagen III-R | CCATAGCTGAACTGAAAACCACC |  |
| M-Tollip-F | CCTCAGCCCCGCTGTAATG |  |
| M-Tollip-R | CAGCATCTTTGTTCCCTCTCTG |  |
| M-histamine1-F | CAAGATGTGTGAGGGGAACAG |  |
| M-histamine1-R | CTACCGACAGGCTGACAATGT |  |
| M-IL-4α-F | TCTGCATCCCGTTGTTTTGC |  |
| M-IL-4α-R | GCACCTGTGCATCCTGAATG |  |
| M-IL13a2-F | ACCGAAATGTTGATAGCGACAG |  |
| M-IL13a2-R | ACAATGCTCTGACAAATGCGTA |  |
| M-CD11-ITGAM-F | ATGGACGCTGATGGCAATACC |  |
| M-CD11-ITGAM-R | TCCCCATTCACGTCTCCCA |  |
| M-CD45-1-PTPRC-F | GTTTTCGCTACATGACTGCACA |  |
| M-CD45-1-PTPRC-R | AGGTTGTCCAACTGACATCTTTC |  |
| M-GAPDH-F | AGGTCGGTGTGAACGGATTTG |  |
| M-GAPDH-R | TGTAGACCATGTAGTTGAGGTCA |  |
| M-Collagen I-F | GTTGCTGCTTGCAGTAACCTT |  |
| M-Collagen I-R | AGGGCCAAGTCCAACTCCTT |  |
| M-Collagen III-F | GGAGCTGGCTACTTCTCGC |  |
| M-Collagen III-R | GGGAACATCCTCCTTCAACAG |  |
| H-Elastin-F | TGTGCCTACGTGGTGACTC |  |
| H-Elastin-R | CGGTACATGATACTTCGGGAAC |  |
| H-MMP1-F | GGGGCTTTGATGTACCCTAGC |  |
| H-MMP1-R | TGTCACACGCTTTTGGGGTTT |  |
| H-MMP2-F | GATACCCCTTTGACGGTAAGGA |  |
| H-MMP2-R | CCTTCTCCCAAGGTCCATAGC |  |
| H-MMP3-F | CTGGACTCCGACACTCTGGA |  |
| H-MMP3-R | CAGGAAAGGTTCTGAAGTGACC |  |
| H-c-Fos-F | CTCGGGCTTCAACGCAGACTA |  |
| H-c-Fos-R | GGAATGAAGTTGGCACTGGAGAC |  |
| H-FOS-B-F | GCTGCAAGATCCCCTACGAAG |  |
| H-FOS-B-F | ACGAAGAAGTGTACGAAGGGTT |  |
| H-JUN-F | TCCAAGTGCCGAAAAAGGAAG |  |
| H-JUN-R | CGAGTTCTGAGCTTTCAAGGT |  |
| H-JUN-B-F | ACAAACTCCTGAAACCGAGCC |  |
| H-JUN-B-R | CGAGCCCTGACCAGAAAAGTA |  |
| H-JUND-F | TCATCATCCAGTCCAACGGG |  |
| H-JUND-R | TTCTGCTTGTGTAAATCCTCCAG |  |
| H-GAPDH-F | GGAGCGAGATCCCTCCAAAAT |  |
| H-GAPDH-R | GGCTGTTGTCATACTTCTCATGG |  |
| H-JNK-F | TGTGTGGAATCAAGCACCTTC |  |
| H-JNK-R | AGGCGTCATCATAAAACTCGTTC |  |
| H-ERK-F | TATCAACCCGTTTGGAGGAAAAG |  |
| H-ERK-R | ATGGAGGCTAAGGTGAACAGT |  |
| H-P38MARK-F | TAACGAAGTTACCAGAGCCCT |  |
| H-P38MARK-R | GTGTTCTTTTTGAACGCCAAGG |  |
| H-PLOD2-F | CATGGACACAGGATAATGGCTG |  |
| H-PLOD2-R | AGGGGTTGGTTGCTCAATAAAAA |  |
| M-MMP-1-F | AACTACATTTAGGGGAGAGGTGT |  |
| M-MMP-1-R | GCAGCGTCAAGTTTAACTGGAA |  |
| M-MMP-3-F | ACATGGAGACTTTGTCCCTTTTG |  |
| M-MMP-3-R | TTGGCTGAGTGGTAGAGTCCC |  |
| M-AP-1-F | TGGTGGCGTTCGTTTCATGT |  |
| M-AP-1-R | GCAGCTCGTAGATGATGACAAA |  |
| M-C-JUN-F | GTCCTCCATAAATGCCTGTTCC |  |
| M-C-JUN-R | GATGCAACCCACTGACCAGAT |  |
| M-C-FOS-F | TTGAGCGATCATCCCGGTC |  |
| M-C-FOS-R | GCGTGAGTCCATACTGGCAAG |  |
| M-JNK-F | ATGGCTGTCGATATTCAACCAG |  |
| M-JNK-R | CCTCTTGGGCATACCCCAC |  |
| M-jun-B-F | TCACGACGACTCTTACGCAG |  |
| M-jun-B-R | CCTTGAGACCCCGATAGGGA |  |
| H-CDKN2A-F | CTTCCTCGGGTGCCGATAC |  |
| H-CDKN2A-R | ACCCCTTCATTGCTACTCGAT |  |
| H-CDKN1A-F | TGTCCGTCAGAACCCATGC |  |
| H-CDKN1A-R | AAAGTCGAAGTTCCATCGCTC |  |
| H-IL-6-F | ACTCACCTCTTCAGAACGAATTG |  |
| H-IL-6-R | CCATCTTTGGAAGGTTCAGGTTG |  |
| H-IL-1β-F | ATGATGGCTTATTACAGTGGCAA |  |
| H-IL-1β-R | GTCGGAGATTCGTAGCTGGA |  |
| H-SERPINE1-F | CCTGGGCACTTACAGGAAGG |  |
| H-SERPINE1-R | GGTCCGATTCGTCGTCAAATAAC |  |
| H-TGFB1-F | GGCCAGATCCTGTCCAAGC |  |
| H-TGFB1-R | GTGGGTTTCCACCATTAGCAC |  |

**Tab. S2** Porosity Measurement of Lyophilized Silk Fibroin Fibers by Mercury Intrusion

| Test Items | SF Freeze-Dried |
| --- | --- |
| Pore Volume | 42.5592 mL/g |
| Total Pore Area | 6.814 m²/g |
| Median pore diameter (volume) | 70,258.57 nm |
| Median pore diameter (area) | 10,360.92 nm |
| Average pore size (4V/A) | 24,984.06 nm |
| Bulk Density | 0.0217 g/mL |
| Apparent Density | 0.2765 g/mL |
| Porosity | 92.1685 % |

**Tab. S3** Temperature measurements of ICR rats during the experimental cycle.

| Group | Temperature(°C) | | | | | Mean ± SEM |
| --- | --- | --- | --- | --- | --- | --- |
| HA Pro-3d | 37.5 | 37.6 | 37.5 | 38.2 | 38.2 | 37.8 |
| HA Pro-1W | 38.9 | 37.9 | 38.0 | 38.1 | 38.5 | 38.28 |
| HA Pro-2W | 39.0 | 38.8 | 39.1 | 39.6 | 38.1 | 38.92 |
| HA Pro-3W | 35.9 | 35.2 | 35.1 | 36.4 | 35.7 | 35.66 |
| HA Pro-4W | 35.1 | 35.0 | 35.4 | 35.1 | 35.0 | 35.12 |
| HA-SF-3d | 38.7 | 38.7 | 38.8 | 38.2 | 38.2 | 38.52 |
| HA-SF-1W | 38.2 | 37.6 | 38.2 | 38.1 | 38.0 | 38.02 |
| HA-SF-2W | 38.8 | 39.0 | 39.1 | 39.6 | 38.1 | 38.92 |
| HA-SF-3W | 34.3 | 35.1 | 36.7 | 35.1 | 34.9 | 35.22 |
| HA-SF-4W | 34.1 | 35.2 | 35.2 | 35.4 | 35.1 | 35 |

**Tab. S4** Body temperature of New Zealand rabbits during the experimental cycle after injection. (℃)

| Group | 0 | 1 | 3 | 5 | 10 | 15 | 20 | 25 | 30d |  |
| --- | --- | --- | --- | --- | --- | --- | --- | --- | --- | --- |
| HA-SF-1 | 37.8 | 38.2 | 38.5 | 38.9 |  |  |  |  |  |  |
| HA-SF-2 | 38.4 | 38.3 | 38.2 | 38.3 | 38.9 | 38.5 |  |  |  |  |
| HA-SF-3 | 38.5 | 38.4 | 38.3 | 38.3 | 38.9 | 38.8 | 38.6 | 38.2 | 38.4 |  |
| Ctrl-1 | 38.4 | 38.4 | 38.7 | 38.3 |  |  |  |  |  |  |
| Ctrl-2 | 38.8 | 38.5 | 38.6 | 38.3 | 38.3 | 38.9 |  |  |  |  |
| Ctrl-3 | 38.4 | 38.3 | 38.3 | 38.4 | 38.6 | 38.5 | 38.7 | 38.2 | 38.8 |  |
| HA Pro-1 | 38.9 | 38.5 | 38.7 | 38.4 |  |  |  |  |  |  |
| HA Pro-2 | 38.3 | 38.6 | 38.4 | 38.6 | 38.3 | 38.4 |  |  |  |  |
| HA Pro-3 | 38.8 | 38.9 | 38.7 | 38.3 | 38.7 | 38.7 | 38.6 | 38.3 | 38.4 |  |
| COL III-1 | 38.2 | 38.4 | 38.9 | 38.7 |  |  |  |  |  |  |
| COL III-2 | 38.5 | 38.3 | 38.3 | 38.4 | 38.3 | 38.3 |  |  |  |  |
| COL III-3 | 38.2 | 38.6 | 38.2 | 38.5 | 38.9 | 38.7 | 38.9 | 38.4 | 38.3 |  |

**Tab. S5** Histologic evaluation system - cell type/response (Phf=per high magnification field of view (400×)).

| Cellular/Tissue Response | Scoring | | | | |
| --- | --- | --- | --- | --- | --- |
|  | 0 | 1 | 2 | 3 | 4 |
| Polymorphonuclear cells | 0 | Very rare，1~5/phf | 5~10/phf | Massive infiltration | Full field of view |
| Lymphocytes | 0 | Very rare，1~5/phf | 5~10/phf | Massive infiltration | Full field of view |
| Plasma cells | 0 | Very rare，1~5/phf | 5~10/phf | Massive infiltration | Full field of view |
| Macrophages | 0 | Very rare，1~5/phf | 5~10/phf | Massive infiltration | Full field of view |
| Megakaryocytes | 0 | Very rare，1~5/phf | 5~10/phf | Massive infiltration | Full field of view |
| Neutrophils | 0 | Very rare，1~5/phf | 5~10/phf | Massive infiltration | Full field of view |
| Necrosis | 0 | Minimal | Slight | Moderate | Severe |

**Tab. S6** Histological Evaluation System - Tissue Response.

| Tissue Response | Scoring | | | | |
| --- | --- | --- | --- | --- | --- |
|  | 0 | 1 | 2 | 3 | 4 |
| Neovascularization | 0 | Slight capillary hyperplasia, focal, 1 to 3 buds | 4 to 7 groups of capillary proliferation, supplemented by fibroblastic structures | Extensive capillary hyperplasia, supplemented by fibroblast structures | Extensive capillary hyperplasia with fibroblast structure |
| Fibrosis | 0 | Restricted areas | Moderately thick areas | Thick areas | Extensive areas |
| Adipose infiltration | 0 | Very few adipocytes with fibrosis | Several layers of adipocytes and fibrosis | Extended and enlarged areas of adipocyte accumulation at the implant site | Area of adipocyte aggregation at the implant site extended and enlarged |
| Inflammatory reaction | 0 | Slight macrophage, neutrophilic infiltrate | Few macrophages, neutrophilic infiltration | More macrophages, neutrophilic infiltration | Extensive macrophage, neutrophilic infiltration |

**Tab. S7** Cellular safety scores of dermatopathologic sections of ICR mice injected with HA-SF injections.

| Cellular/Tissue Response | A1 | A2 | A3 | A4 | A5 | B1 | B2 | B3 | B4 | B5 | CTRL |
| --- | --- | --- | --- | --- | --- | --- | --- | --- | --- | --- | --- |
| Polymorphonuclear cells | 1 | 1 | 1 | 2 | 1 | 1 | 1 | 1 | 1 | 1 | 1 |
| Lymphocytes | 1 | 1 | 1 | 1 | 1 | 1 | 1 | 1 | 1 | 1 | 1 |
| Plasma cells | 0 | 0 | 0 | 0 | 0 | 0 | 0 | 0 | 0 | 0 | 0 |
| Macrophages | 1 | 1 | 2 | 2 | 2 | 1 | 1 | 1 | 1 | 1 | 1 |
| Megakaryocytes | 0 | 0 | 1 | 0 | 0 | 0 | 0 | 0 | 0 | 0 | 0 |
| Neutrophils | 2 | 1 | 1 | 1 | 1 | 1 | 1 | 1 | 1 | 1 | 2 |
| Necrosis | 0 | 0 | 0 | 0 | 0 | 0 | 0 | 0 | 0 | 0 | 0 |

**Tab. S8** Tissue Safety Score on Skin Pathology Sections of ICR Mouse Injected with Hydrafacial Injection.

| Tissue Response | A1 | A2 | A3 | A4 | A5 | B1 | B2 | B3 | B4 | B5 | CTRL |
| --- | --- | --- | --- | --- | --- | --- | --- | --- | --- | --- | --- |
| Neovascularization | 0 | 0 | 0 | 0 | 0 | 0 | 0 | 0 | 0 | 0 | 0 |
| Fibrosis | 0 | 0 | 0 | 0 | 0 | 0 | 0 | 0 | 0 | 0 | 0 |
| Adipose infiltration | 0 | 2 | 1 | 2 | 1 | 1 | 1 | 2 | 1 | 1 | 1 |
| Inflammatory reaction | 3 | 1 | 2 | 1 | 1 | 2 | 2 | 2 | 2 | 1 | 2 |

**Tab. S9** Cellular safety scores on dermatopathology sections of New Zealand rabbits injected with hydrafacial injections.

| Cellular/Tissue Response | CTRL | HA Pro | Col III | HA-SF |
| --- | --- | --- | --- | --- |
| Polymorphonuclear cells | 1 | 2 | 1 | 2 |
| Lymphocytes | 0 | 2 | 2 | 2 |
| Plasma cells | 0 | 1 | 1 | 1 |
| Macrophages | 1 | 1 | 1 | 1 |
| Megakaryocytes | 0 | 1 | 1 | 1 |
| Neutrophils | 0 | 0 | 0 | 0 |
| Necrosis | 0 | 0 | 0 | 0 |

**Tab. S10** Tissue safety scores on dermatopathologic sections of New Zealand rabbits injected with hydrafacial injections.

| Tissue Response | CTRL | HA Pro | Col III | HA-SF |
| --- | --- | --- | --- | --- |
| Neovascularization | 0 | 0 | 0 | 0 |
| Fibrosis | 0 | 0 | 0 | 0 |
| Adipose infiltration | 0 | 0 | 0 | 0 |
| Inflammatory reaction | 0 | 2 | 1 | 1 |

**Tab. S11** Hematological Parameters of Male Rats Following HA-SF Injection

| Item | Unit | Experimental Group | Control Group | P-value |
| --- | --- | --- | --- | --- |
| White Blood Cell Count (WBC) | (10^9^/L) | 3.71±1.41 | 4.28±0.94 | 0.303 |
| Granulocyte Count (Gran#) | (10^9^/L) | 0.91±0.35 | 1.06±0.27 | 0.282 |
| Lymphocyte Count (LY#) | (10^9^/L) | 2.35±1.03 | 2.75±0.78 | 0.33 |
| Monocyte Count (Mid#) | (10^9^/L) | 0.38±0.35 | 0.38±0.3 | 0.989 |
| Eosinophil Count (EOS#) | (10^9^/L) | 0.08±0.03 | 0.08±0.03 | 0.891 |
| Basophil Count (BASO#) | (10^9^/L) | 0 | 0±0 | 1 |
| Neutrophil Percentage (NEUT%) | (%) | 26.4±8.35 | 26.04±8 | 0.923 |
| Lymphocyte Percentage (LYMPH%) | (%) | 62.18±5.88 | 63.62±5.59 | 0.582 |
| Monocyte Percentage (Mon%) | (%) | 9.09±6.03 | 8.33±5.07 | 0.764 |
| Eosinophil Percentage (EOS%) | (%) | 2.21±0.62 | 1.94±0.91 | 0.447 |
| Basophil Percentage (BASO%) | (%) | 0.12±0.13 | 0.07±0.08 | 0.322 |
| Red Blood Cell Count (RBC) | (10^12^/L) | 7.67±1.78 | 8.39±0.98 | 0.275 |
| Hemoglobin (HGB) | (g/L) | 148.8±29.29 | 160.3±13.39 | 0.28 |
| Hematocrit (HCT) | (%) | 43.62±10.18 | 47.38±5.1 | 0.31 |
| Mean Corpuscular Volume (MCV) | (fL) | 56.77±1.83 | 56.52±0.75 | 0.697 |
| Mean Corpuscular Hemoglobin (MCH) | (pg) | 19.64±1.11 | 19.18±0.66 | 0.277 |
| Mean Corpuscular Hemoglobin Concentration (MCHC) | (g/L) | 346.2±26.39 | 340.2±10.96 | 0.519 |
| Red Cell Distribution Width Standard Deviation (RDW-SD) | (fL) | 26.06±2.61 | 26.46±3.25 | 0.765 |
| Platelet Count (PLT) | (10^9^/L) | 912.1±264.01 | 969.8±51.25 | 0.513 |
| Mean Platelet Volume (MPV) | (fL) | 6.45±0.25 | 6.36±0.36 | 0.529 |
| Alanine Transaminase (ALT) | (U/L) | 23.6±8.42 | 16.4±4.09 | 0.030* |
| Aspartate Transaminase (AST) | (U/L) | 236.61±64.73 | 198.9±57.44 | 0.185 |
| Alkaline Phosphatase (ALP) | (U/L) | 193.7±88.67 | 180.3±99.68 | 0.754 |
| Total Protein (TP) | (g/L) | 63.51±7.82 | 65.21±5.63 | 0.584 |
| Albumin (ALB) | (g/L) | 33.98±5.6 | 34.48±3.18 | 0.809 |
| Total Bilirubin (T-Bil) | (μmol/L) | 0.37±0.16 | 0.28±0.15 | 0.213 |
| Globulin (GLB) | (g/L) | 29.6±2.37 | 30.7±3.2 | 0.394 |
| Albumin/Globulin Ratio (A/G) | / | 1.16±0.13 | 1.13±0.12 | 0.641 |
| Urea (UREA) | (mmol/L) | 7.46±1.38 | 7.8±1.83 | 0.65 |
| Creatinine (CREA-J) | (μmol/L) | 34±6.85 | 39.2±7.64 | 0.126 |
| Glucose (Glu) | (mmol/L) | 13.07±2 | 12.82±2.66 | 0.819 |
| Total Cholesterol (TC) | (mmol/L) | 1.57±0.33 | 1.56±0.48 | 0.94 |
| Triglycerides (TG) | (mmol/L) | 1.98±1.04 | 1.66±1.19 | 0.529 |
| Potassium (K) | (mmol/L) | 5.78±0.51 | 5.71±0.52 | 0.777 |
| Sodium (Na) | (mmol/L) | 174.78±3.72 | 175.54±2.51 | 0.599 |
| Calcium (Ca) | (mmol/L) | 1.99±0.1 | 2.06±0.21 | 0.36 |
| Chloride (CI) | (U/L) | 91.89±2.29 | 94.63±2.6 | 0.022* |
| Phosphorus (P) | (mmol/L) | 1.69±0.5 | 1.81±0.52 | 0.61 |
| Gamma-Glutamyltransferase (y-GT) | (U/L) | 1.2±0.92 | 1.3±1.06 | 0.824 |
| Activated Partial Thromboplastin Time (APTT) | (s) | 23.98±0.89 | 25.69±1.62 | 0.009* |
| Prothrombin Time (PT) | (s) | 15.87±1.28 | 40.29±53.92 | 0.186 |

**Tab. S12** Organ Weight Statistics (Males) (Unit: g)

| Organs | Experimental Group | Control Group | P-value |
| --- | --- | --- | --- |
| Heart | 1.54±0.2 | 1.51±0.24 | 0.802 |
| Liver | 17.04±2.39 | 17.42±1.69 | 0.686 |
| Spleen | 0.71±0.06 | 0.84±0.13 | 0.015* |
| Lungs | 2.23±0.3 | 2.9±0.3 | 0.000* |
| Both kidneys | 3.28±0.31 | 3.23±0.54 | 0.814 |
| Brain | 2.02±0.13 | 1.98±0.1 | 0.513 |
| Testicles | 3.58±0.32 | 3.22±0.33 | 0.022 |
| Epididymis | 1.41±0.14 | 1.42±0.09 | 0.809 |
| Thymus | 0.65±0.13 | 0.88±0.23 | 0.013* |
| Adrenal glands | 0.05±0.01 | 0.05±0.01 | 0.845 |

**Tab. S13** The comparison between the UVB and the CTRL group at 30 day.

| UVB Group (Model Group) | CTRL Group |
| --- | --- |
| 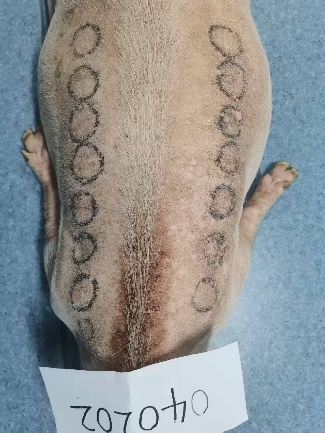 | 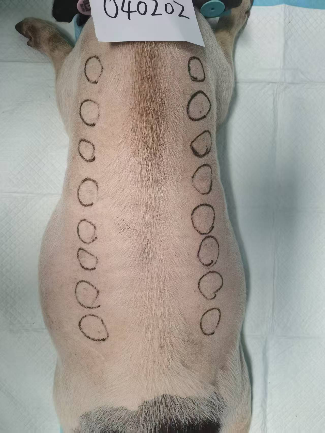 |
| Bama pig in the model group exhibited uneven skin tone, capillary dilation, and occasional erythema. The skin surface appeared rough and thickened, with scaling and a leathery appearance, alongside a noticeable loss of elasticity. | The skin of bama pig in the CTRL group is tender with an even complexion, showing no telangiectasia or localized erythema. The skin exhibits good elasticity. |

**Methods：**

Hematoxylin and Eosin (H&E) Staining: After fixation of skin tissues in paraformaldehyde, samples were dewaxed and rehydrated through xylene I–III (5 min each), followed by absolute ethanol I–II, 95% ethanol I–II, 90%, 80%, 70% ethanol, and rinsed in tap water. Sections were stained with Harris hematoxylin for 5 minutes, rinsed in water, differentiated in acid alcohol, blued in ammonia water, and washed again. Eosin staining was performed, followed by graded ethanol dehydration and xylene clearing. Sections were sealed with neutral resin and observed under a light microscope.

Masson's Trichrome Staining: After dewaxing to water as described above, sections were incubated in Bouin's solution at 37°C for 2 hours. After rinsing under running water until yellow color disappeared, sections were stained with Weigert’s iron hematoxylin, followed by Biebrich scarlet-acid fuchsin, phosphomolybdic acid, and aniline blue. Sections were then washed with distilled water, treated with 0.2% acetic acid (5 S × 3), dehydrated in absolute ethanol, cleared in xylene, and mounted with neutral resin.

Sirius Red Staining: Sections were dewaxed and rehydrated as described, then stained in Sirius Red solution for 1 minute. After rinsing in distilled water, sections were dehydrated in absolute ethanol (1 min × 2), cleared in xylene (5 min × 3), and mounted with neutral resin.

IF Staining: Skin samples were fixed for over 48 h, rinsed in PBS, dehydrated in graded ethanol, embedded in paraffin, and sectioned at 4 μm. After baking at 60°C for 4 hours, slides were dewaxed to water, and antigen retrieval was performed using EDTA (pH 9.0) in a microwave. Sections were washed with PBS (5 min × 3), incubated with 3% H₂O₂ for 30 minutes, followed by blocking with goat serum for 20 minutes. Primary antibodies (1:8000) were applied overnight at 4°C. After PBS washing, secondary antibodies (1:500) were applied for 1 hour at 37°C. Sections were then developed with DAB (1 min 20 s), rinsed in PBST to stop the reaction, counterstained with Mayer's hematoxylin for 1 minute, dehydrated, cleared, and mounted.

In vivo Efficacy Assessment in Rabbits: Skin hydration, elasticity, and collagen content were evaluated using a skin analysis system (DermaLab Combo 4, Cortex) on days 0–30 after intradermal injection in New Zealand rabbits. Hydration: Measured by conductometry based on differences in dielectric constants between water and other skin components, expressed in microsiemens (µS). Each site was measured in triplicate. Hydration increase rate = (measured hydration − baseline hydration) / baseline hydration × 100%. Elasticity: Assessed via negative pressure suction and optical detection. The system applied vacuum suction to the skin and measured deformation depth (MPa) to determine elasticity.

Elasticity Parameters of Skin: Ue (Immediate distension): Represents the initial elastic deformation of the skin under vacuum pressure, showing a linear relationship between deformation amplitude and applied force. Uv (Viscoelastic/plastic distension): Refers to delayed deformation after the initial elastic response when vacuum is sustained; the deformation is no longer linearly related to the applied force. Uf (Final distension): The total deformation of the skin, including both elastic and plastic components (Uf = Ue + Uv). Ur (Immediate retraction): Denotes the initial elastic recovery of the skin after vacuum release, proportional to the deformation during Ue. Ua (Final retraction): The total retraction observed from vacuum release to the start of the next suction cycle, indicating full viscoelastic recovery.

Quantitative Parameters: Young’s modulus (E): Represents the stiffness of the skin. It is calculated based on the distance stretched under a predefined vacuum pressure. Higher values indicate lower deformability. Retraction time (R): The time required for the skin to retract from its peak distension to 33% of that value. Lower values indicate greater firmness. Viscoelasticity (VE): A composite of immediate and delayed elasticity. Higher values reflect better skin firmness.

R2 = Ua / Uf: Indicates the ratio of total retraction to maximum deformation. Values closer to 1 denote higher elasticity. R5 = Ur / Ue: Reflects the proportion of elastic recovery to elastic distension in the first test cycle. Higher values indicate better skin elasticity. R6 = Uv / Ue: Indicates the ratio of plastic to elastic deformation. Lower values reflect better elasticity. R7 = Ur / Uf: Measures the proportion of immediate retraction to total deformation. Values approaching 1 suggest superior elastic recovery.

Principle of Collagen Measurement: Collagen content was assessed using high-frequency skin ultrasound based on echo intensity. This method evaluates the density and thickness of the epidermis, dermis, and subcutaneous layers. The mean echo intensity quantitatively reflects collagen content; higher values indicate greater collagen density.

Color-coded echo images represent varying tissue densities: Black/green areas correspond to low-density regions (e.g., fat, fluid, blood). Yellow/white areas indicate high-density regions (e.g., collagen, elastin). The diversity of color patterns also reflects structural complexity of the tissue. This non-invasive method enables assessment of skin aging, photoaging, sensitivity, vascular lesions, scarring, wound healing, and tumors.
